# The nature and chronology of human occupation at the Galerías Bajas, from Cueva de Ardales, Malaga, Spain

**DOI:** 10.1371/journal.pone.0266788

**Published:** 2022-06-01

**Authors:** José Ramos-Muñoz, Pedro Cantalejo, Julia Blumenröther, Viviane Bolin, Taylor Otto, Miriam Rotgänger, Martin Kehl, Trine Kellberg Nielsen, Mar Espejo, Diego Fernández-Sánchez, Adolfo Moreno-Márquez, Eduardo Vijande-Vila, Lidia Cabello, Serafín Becerra, África Pitarch Martí, José A. Riquelme, Juan J. Cantillo-Duarte, Salvador Domínguez-Bella, Pablo Ramos-García, Yvonne Tafelmaier, Gerd-Christian Weniger

**Affiliations:** 1 Department of History, Geography and Philosophy, University of Cadiz, Cadiz, Spain; 2 Cueva de Ardales, Ardales, Malaga, Spain; 3 Ayuntamiento de Ardales, Ardales, Malaga, Spain; 4 Institute of Prehistoric Archaeology, University of Erlangen, Erlangen, Germany; 5 Museum der Stadt Ulm, Ulm, Germany; 6 Institute of Prehistoric Archaeology, University of Cologne, Cologne, Germany; 7 Commission for Archeology of Non-European Cultures, Bonn, Germany; 8 Institute of Geography, University of Cologne, Cologne, Germany; 9 School of Culture and Society, Department of Archeology and Heritage Studies, Aarhus, Denmark; 10 ArdalesTur, Ardales, Malaga, Spain; 11 Department of Geography, History and Humanities, University of Almeria, Almeria, Spain; 12 Dolmens of Antequera Archaeological Ensemble, Antequera, Malaga, Spain; 13 Secondary School Itaba, Teba, Malaga, Spain; 14 Departament d’Arts i Conservació-Restauració, Facultat de Belles Arts, Universitat de Barcelona, Barcelona, Spain; 15 Department of History, University of Cordoba, Cordoba, Spain; 16 Earth Sciences Department, Campus Rio San Pedro, Universityt of Cadiz, Puerto Real, Cadiz, Spain; 17 School of Dentistry, University of Granada, Granada, Spain; 18 State Office for Cultural Heritage Baden-Wuerttemberg, Esslingen, Germany; University at Buffalo - The State University of New York, UNITED STATES

## Abstract

The Cueva de Ardales is a hugely important Palaeolithic site in the south of the Iberian Peninsula owing to its rich inventory of rock art. From 2011–2018, excavations were carried out in the cave for the first time ever by a Spanish-German research team. The excavation focused on the entrance area of the cave, where the largest assemblage of non-figurative red paintings in the cave is found. A series of 50 AMS dates from the excavations prove a long, albeit discontinuous, occupation history spanning from the Middle Palaeolithic to the Neolithic. The dating of the Middle Palaeolithic layers agrees with the U/Th dating of some red non-figurative paintings in the entrance area. In addition, a large assemblage of ochre lumps was discovered in the Middle Palaeolithic layers. Human visits of the cave in the Gravettian and Solutrean can be recognized, but evidence from the Aurignacian and Magdalenian cannot be confirmed with certainty. The quantity and nature of materials found during the excavations indicate that Cueva de Ardales was not a campsite, but was mainly visited to carry out non-domestic tasks, such as the production of rock art or the burial of the dead.

## Introduction

**Fig 1:**
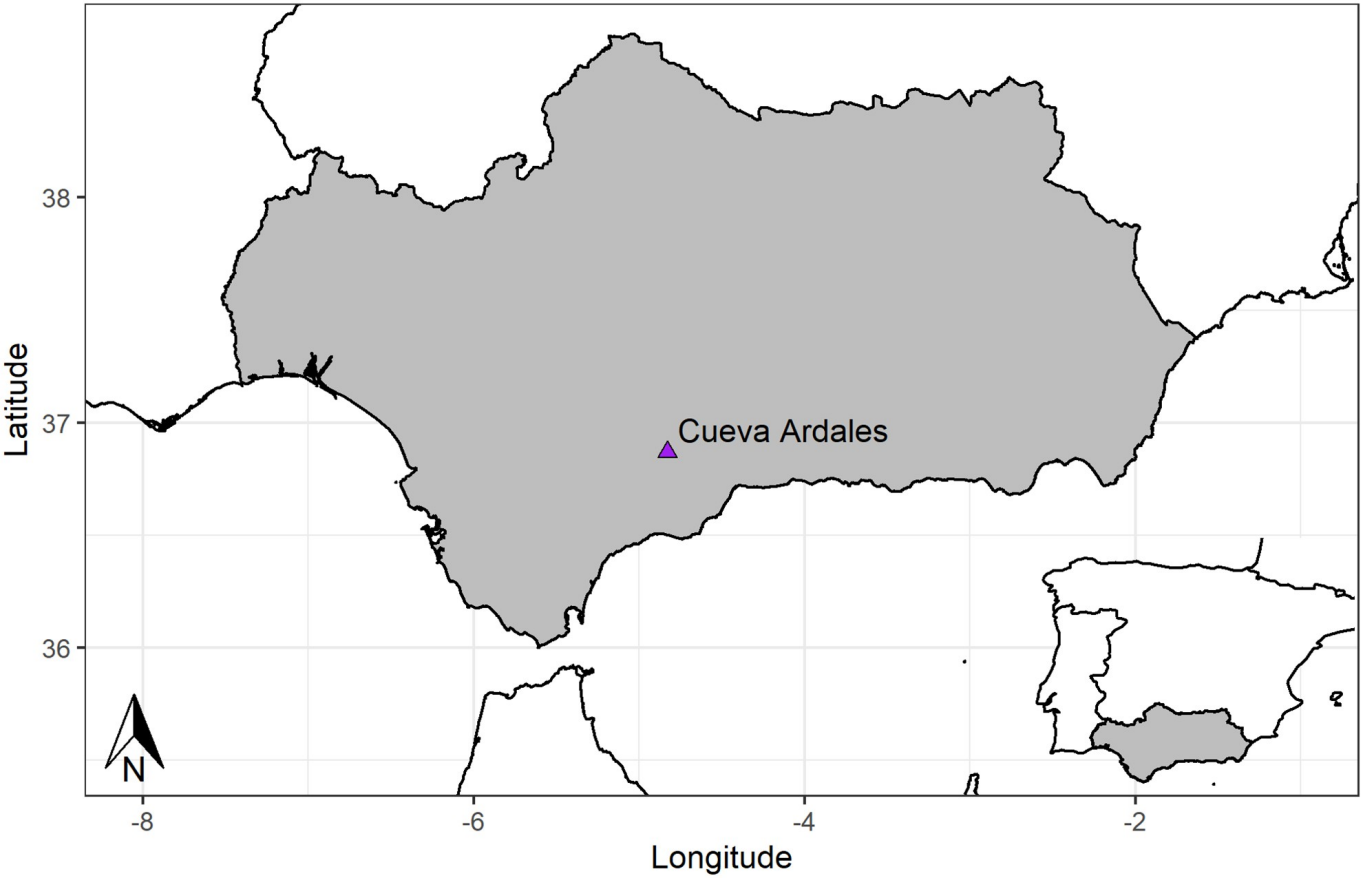
Geographical position of Ardales on the Iberian Peninsula. In grey Region Autonoma de Andalucia.

Cueva de Ardales in Málaga, Spain, is the most outstanding cave with Palaeolithic rock art in southern Iberia. The cave (UTM 337.110/4.082.540) is located near the village of Ardales, in a mountain know as Cerro de la Calinoria, at 565 m a.s.l. and at about 50 km north of the Mediterranean coast (Fig 1). It was discovered in 1821 after an earthquake exposed a cave entrance previously sealed by colluvial deposits. From 1852 on, the cave opened to local tourism although it was not until 1918 that Henri Breuil recognized the Palaeolithic rock art [1]. In the following decades, the cave was paid no further research attention. Research resumed in 1990 [2], leading to a full inventory of the rock art present at the cave [3].

The site is a multi-branched karstic system that is divided into five areas: Area I (Sala del Saco), area II (Sala de las Estrellas), area III (Galería de los Laberintos), area IV (Calvario) and area V (Galerías Altas) [3–7]. Area V was discovered in 1981 by speleologists, comprising a separate cave system situated above the main cave area. Today the Galerías Altas are accessible from the Galerías Bajas only by a narrow fissure that opens in a wall about 18 m above ground. The natural entrance to the Galerías Altas was probably sealed by a landslide in the late Holocene. Coring outside the cave provided evidence for this entrance [7]. This part of the cave has not yet been analysed systematically,but burials from the Copper Age and rock art which, based on stylistic patterns, is dated to the Upper Palaeolithic have been recorded during brief expeditions into the Galerías Altas [7, 8].

The cave contains over 1,000 paintings and engravings found on a wide variety of surfaces including walls, ceilings, ground rocks, speleothems and collapsed blocks. They are mainly dated to the Upper Palaeolithic, although the recent U/Th dating of carbonate crusts on abstract red depictions revealed that some of them are of Neanderthal authorship [9]. Throughout the entrance area, rock art is found in the form of abstract depictions of varying size and shape and hand-stencils. Except for two black negative hand-stencils located in the Sala de las Estrellas, the other paintings were executed in red colour. This distinguishes the entrance area from the interior zones of the cave, where over 90% of the animal representations were documented [3].

Owing to this rich inventory of rock art, Cueva de Ardales represents a key Palaeolithic site in southern Iberia. Despite the fact that it was discovered over 200 years ago the nature of human occupation and the use of the cave apart from the production of rock art was unknown until recently, as no excavations had previously been carried out. In order to gain a better understanding of the human activities, that took place in the cave, their chronology and their potential relationship to the rock art, a Spanish-German team lead by two of the authors (JRM and GCW) conducted excavations in the cave between 2011 and 2018. This article presents the main results of this research program.

### Methodology description of the excavations and their results

#### Location and description of the excavated areas

The cave is currently accessible thanks to Doña Trinidad Grund, its first owner, who had built a staircase in the 19^th^ century.

The excavations in Cueva de Ardales are carried out within the framework of a General Research Project authorised by the Ministry of Culture and Historical Heritage of the Andalusian Regional Government under the title: Prehistoric societies (from the Middle Palaeolithic to the Final Neolithic) in the Cueva de Ardales and the Sima de las Palomas Cave in Teba—Malaga, Spain. Geoarchaeological, chronological and environmental studies. Reference: SIDPH/DI: 201564100003000.

**Fig 2:**
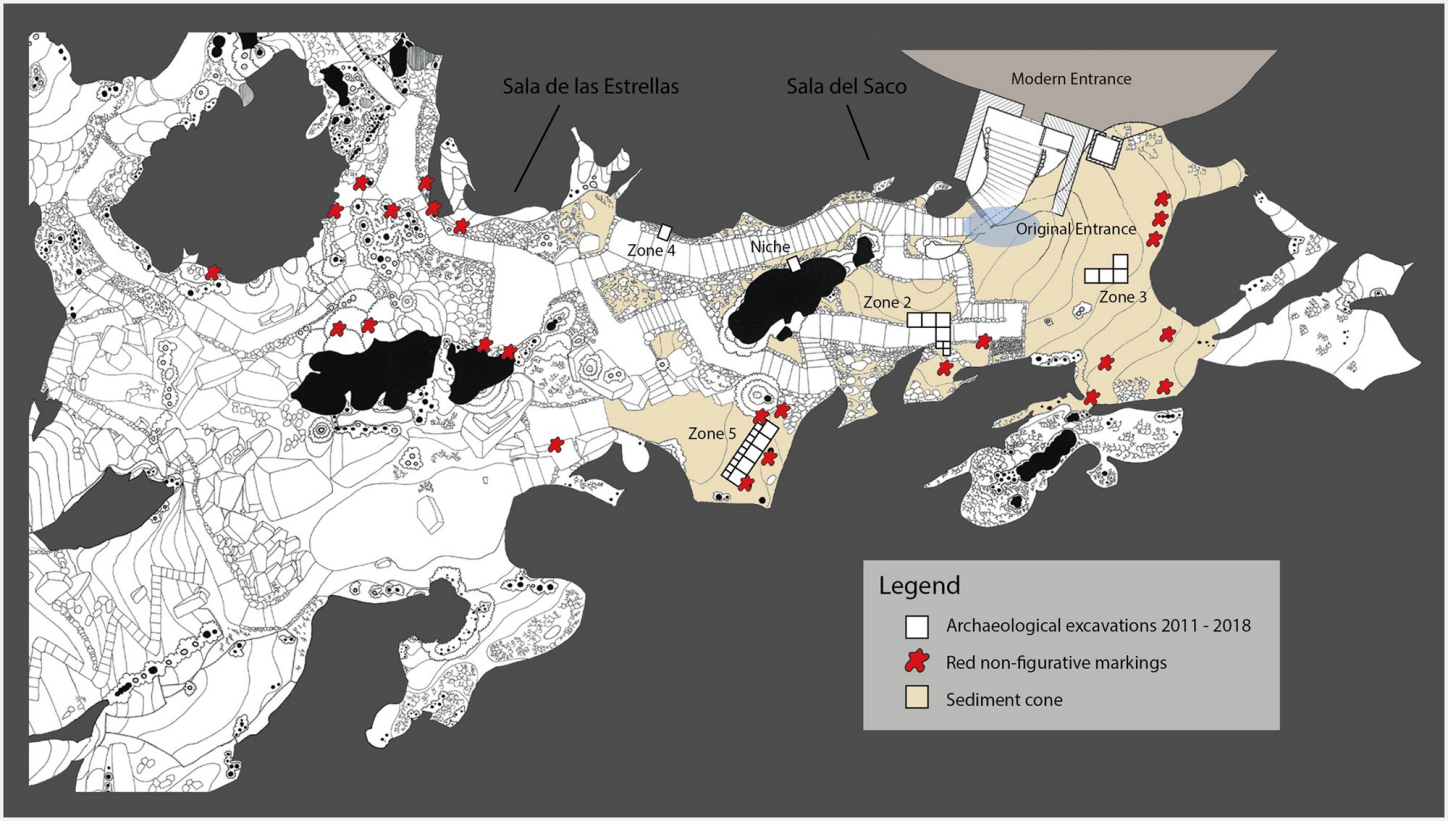
The entrance area of the Cueva de Ardales and the excavation areas.

The stairs were cut into a steep sediment cone that stretches over 20 m from the opening of the cavity via the Sala del Saco down to the Sala de las Estrellas. A total of five zones, located between Sala del Saco and Sala de las Estrellas, were investigated (Fig 2). Zone 1 can be ignored here as it yielded no results owing to the fact that the existing sediments were thoroughly disturbed. Here we report on excavation areas 2–5 and a niche used as a burial place in the Neolithic. The cone is the result of frequent sediment depositions from the slope above the cave entrance. Before the cave’s discovery it was completely covered by a flowstone that protected the sediments underneath. This layer was partly destroyed during the construction of the stairs and footpaths in the 19^th^ century. The steep topography of the entrance makes it unsuitable for human occupation. The only areas where flat surfaces might have been available are the ones in front of the cave entrance and at the foot of the cone.

**Fig 3:**
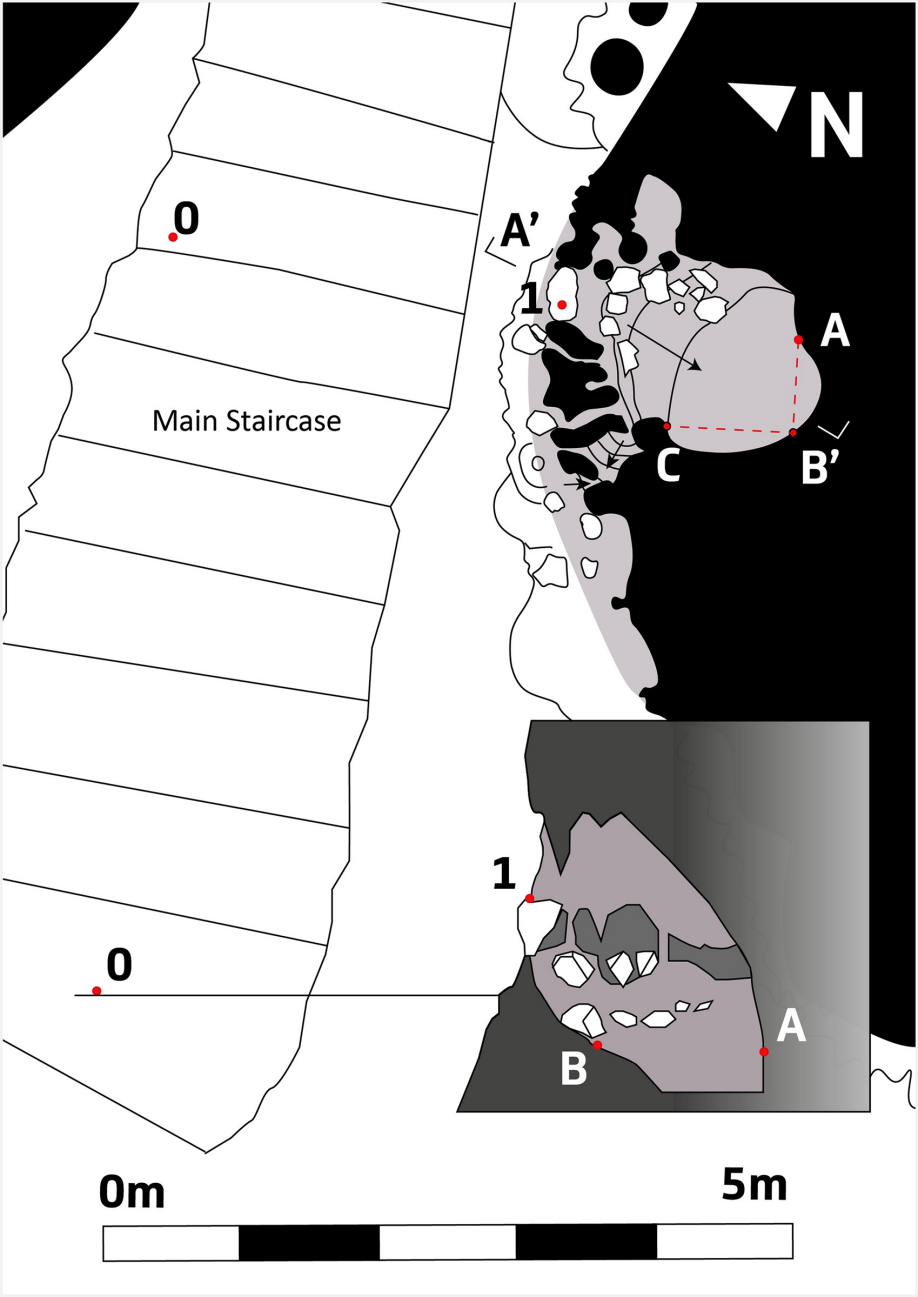
Location of a niche with human remains in speleothem pillars along the main stair case.

Along the main staircase, small niches in the massive speleothems yielded pottery fragments and a human remain (Fig 3). The latter is a mandible fragment from a twelve-year-old male. Radiocarbon dates the mandible to the Neolithic (Table 1) which agrees with the chronology of an associated fragment of decorated pottery. Other human remains were found on the surface of the cone area, some of which were encrusted in the flowstone [8–10].

**Table 1:** Radiocarbon dates for human occupations in the Galerías Bajas of Cueva de Ardales.

Lab number	Context	Layer	BP	±	calBP 68%	± 68%	calBP 95%	Pretreatment	Material
OxA-35394	Sala Estrellas	Calcified surface on a rock ledge	366	28	405	70	550–270		Textile (rope)
COL3066.1.1	Sala Estrellas	Stationary lamp	4107	41	4664	108	4880–4440	AAA	Charcoal
COL3065.1.1	Sala Estrellas	Stationary lamp	9886	52	11311	68	11430–11190	AAA	Charcoal
MAMS-48675	Galerias Bajas	Staircase	6236	24	7178	47	7280–7080		Tooth roots
MAMS-48673	Zone 2	Concreted	5999	23	6837	40	6920–6760		Tooth fragments
MAMS-48674	Zone 2	Concreted	6000	24	6838	40	6920–6760		Tooth roots
COL1636.1.1	Zone 2	Between flowstone 1 and 2	3885	36	4320	64	4440–4200	AAA	Charcoal
COL1637.1.1	Zone 2	2 or 3	3621	35	3935	50	4030–3830	AAA	Charcoal
COL1640.1.1	Zone 2	2 or 3	3718	40	4064	65	4200–3920	AAA	Charcoal
COL5240.1.1	Zone 2	7	6148	45	7053	76	7210–6890	AAA	Charcoal
COL5250.1.1	Zone 2	11	3653	39	3986	70	4130–3850	AAA	Charcoal
COL5245.1.1	Zone 2	11	6114	44	7019	88	7200–6840	A	Charcoal
COL5238.1.1	Zone 2	11	6162	44	7064	69	7200–6920	AAA	Charcoal
COL5239.1.1	Zone 2	11	6282	44	7209	43	7290–7130	AAA	Charcoal
COL5251.1.1	Zone 2	11	6389	46	7334	58	7450–7210	AAA	Charcoal
COL5249.1.1	Zone 2	11	12390	61	14485	184	14850–14090	AAA	Charcoal
COL1639.1.1	Zone 2	13/14	15945	60	19268	146	19500–18980	Short AAA	Charcoal
COL4583.1.1	Zone 2	15	20673	86	24827	217	25310–24470	A	Charcoal
COL4584.1.1	Zone 2	16	24639	108	28774	158	28940–28420	A	Charcoal
COL2011.1.1	Zone 3	Profile E2/F2	5562	48	6352	43	6430–6270		Bone
COL5493.1.1	Zone 3	Surface (underneath large rock)	6138	42	7046	79	7210–6890	AAA	Charcoal
COL5497.1.1	Zone 3	C1.2	6145	52	7047	82	7210–6890	AAA	Charcoal
COL5495.1.1	Zone 3	C1.2	6179	43	7078	66	7220–6940	AAA	Charcoal
COL5494.1.1	Zone 3	C1.2	6193	44	7091	70	7230–6950	AAA	Charcoal
COL5496.1.1	Zone 3	C1.2	6198	52	7098	79	7260–6940	AAA	Charcoal
COL5247.1.1	Zone 3	Between flowstone layers	27782	156	31765	191	31850–31210	AAA	Charcoal
COL5248.1.1	Zone 3	K	50041	1103	52950	1710		AAA	Charcoal
COL1643.1.1	Zone 3	3	51914	2324	54815	2708		Short AAA	Charcoal
COL1644.1.1	Zone 3	3	53071	2676	55986	3033		AAA	Charcoal
COL4581.1.1	Zone 3	4	>58000	0				A	Charcoal
COL4582.1.1	Zone 3	5	>58000	0				A	Charcoal
COL1641.1.1	Zone 4	2	3747	40	4101	79	4260–3940	AAA	Charcoal
COL1642.1.1	Zone 4	3	3715	40	4061	65	4180–3940	AAA	Charcoal
COL3067.1.1	Zone 5	Surface	267	35	347	71	500–220	AAA	Charcoal
COL5244.1.1	Zone 5	Pertubation	20360	112	24505	176	24910–24110	A	Charcoal
COL5243.1.1	Zone 5	1a	6156	46	7059	73	7200–6920	A	Charcoal
COL4449.1.1	Zone 5	2a	3945	40	4387	74	4530–4250	A	Charcoal
COL5246.1.1	Zone 5	2	23168	115	27401	122	27660–27260	A	Charcoal
COL3654.1.1	Zone 5	2	26024	114	30304	151	30750–29830	AAA	Charcoal
COL4450.1.1	Zone 5	2	26465	160	30711	181	31020–30460	A	Charcoal
COL5241.1.1	Zone 5	2	27010	147	31153	134	31240–30880	A	Charcoal
COL5242.1.1	Zone 5	2	27028	150	31166	135	31250–30890	AAA	Charcoal
COL4446.1.1	Zone 5	2	28277	207	32346	319	32910–31430	A	Charcoal
COL4445.1.1	Zone 5	2	28443	196	32564	336	33120–31600	A	Charcoal
COL4451.1.1	Zone 5	2 or 3	22146	115	26421	184	26700–26020	A	Charcoal
COL3655.1.1	Zone 5	3a	31315	216	35732	343	35710–34710	AAA	Charcoal
COL3653.1+2HA	Zone 5	3b or 4	31519	221	35956	300	35940–34860	AAA	Charcoal
COL4447.1.1	Zone 5	4	40180	634	43402	553	44920–42720	A	Charcoal
COL4448.1.1	Zone 5	4	40514	661	43619	601	45250–42890	A	Charcoal
COL4444.1.1	Zone 5	4	43697	945	46374	1137		A	Charcoal

**Fig 4:**
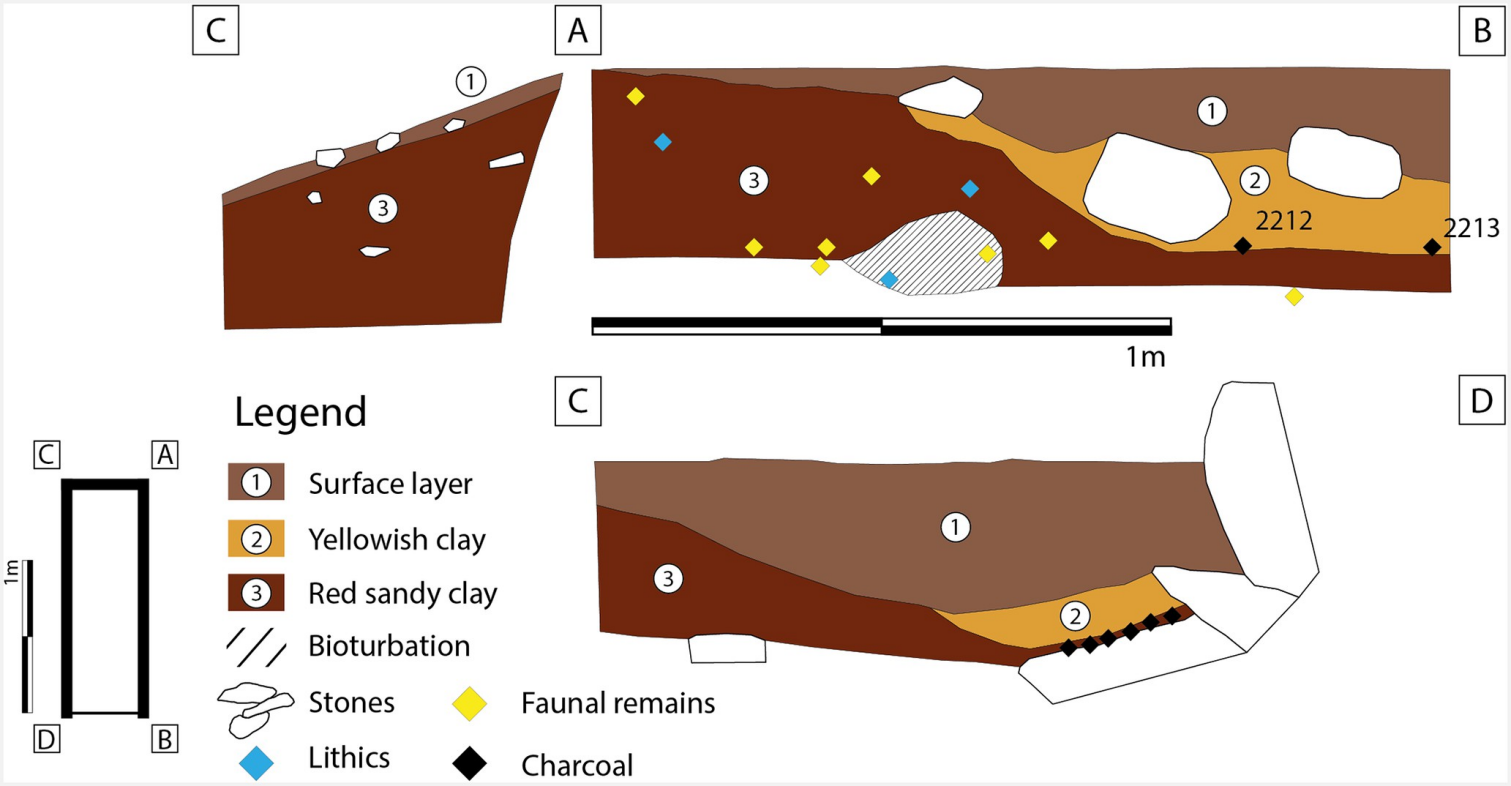
Excavation plan and sections: Zone 4.

**Fig 5:**
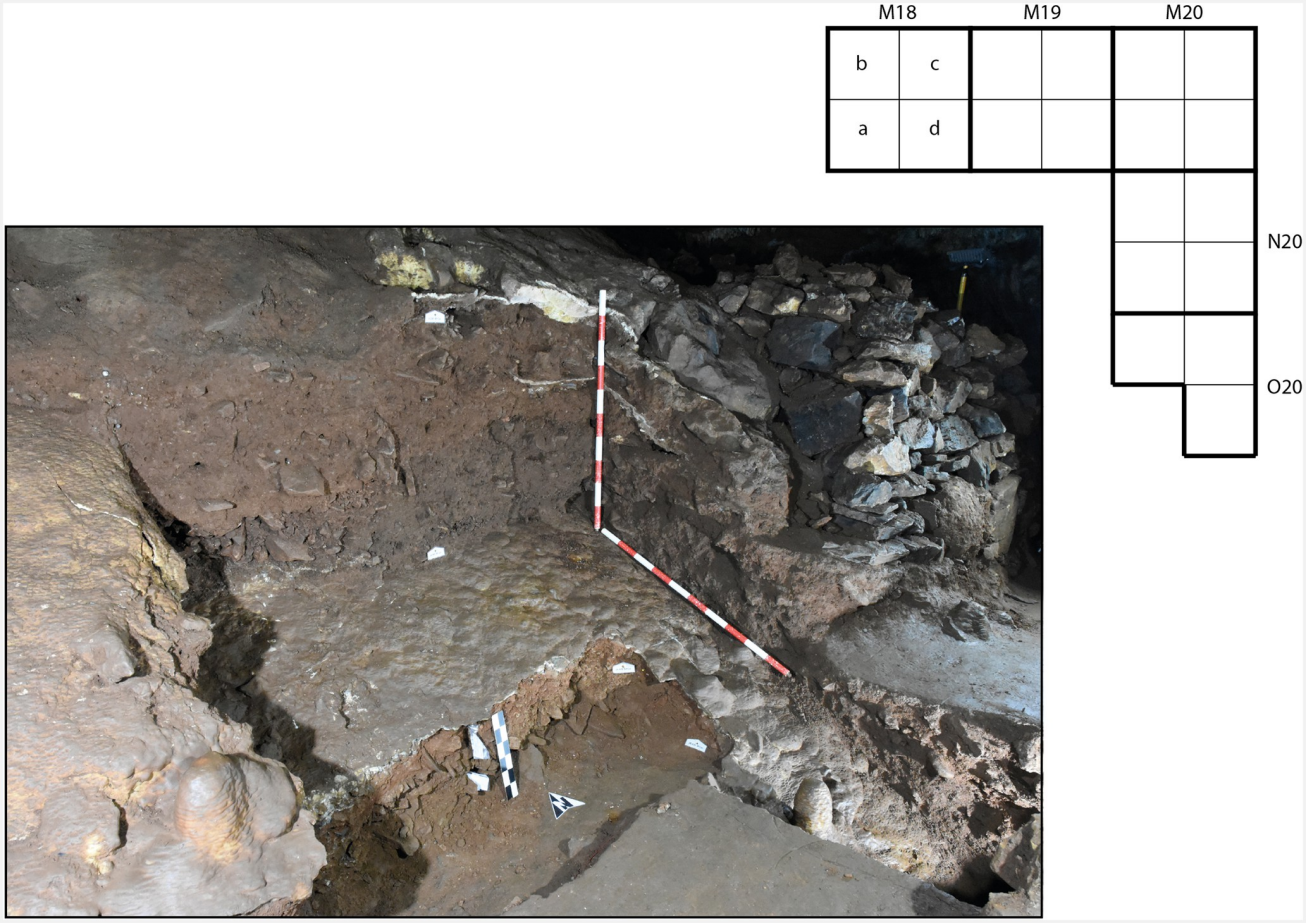
Post-excavation view of zone 2. The plan shows the arrangement of the excavation squares.

Further down the slope a small section was opened on the staircase in front of the cave wall. This area belongs to the Sala de las Estrellas (sector II.C) [3] and was called **zone 4**. In the narrow trench in zone 4 (Fig 4) (1.5 m long and 0.50 m wide) three layers of sediment could be identified. Layers 2 and 3 yielded, two flakes, one of which was heavily burned, over 40 faunal remains, and several large pieces of charcoal (Fig 5). Most of the faunal remains belong to *Oryctolagus*, along with single finds of *Cervus* and *Ibex*, undetermined bird remains and one turtle fragment. The presence of a carnivore is documented by *Lynx* remains (Table 5). Although the sediment of layer 2 is light red in colour and yielded an important accumulation of charcoal together with a burned flake, micromorphological analysis does not suggest that this fill was subject to high temperatures. Therefore, the presence of a hearth cannot be confirmed. Two radiocarbon dates from charcoal remains found in layer 2 and the interface between layers 2 and 3 yielded ages of 4,101 ± 79 calBP and of 4,061 ± 65 calBP (Table 1).

**Fig 6:**
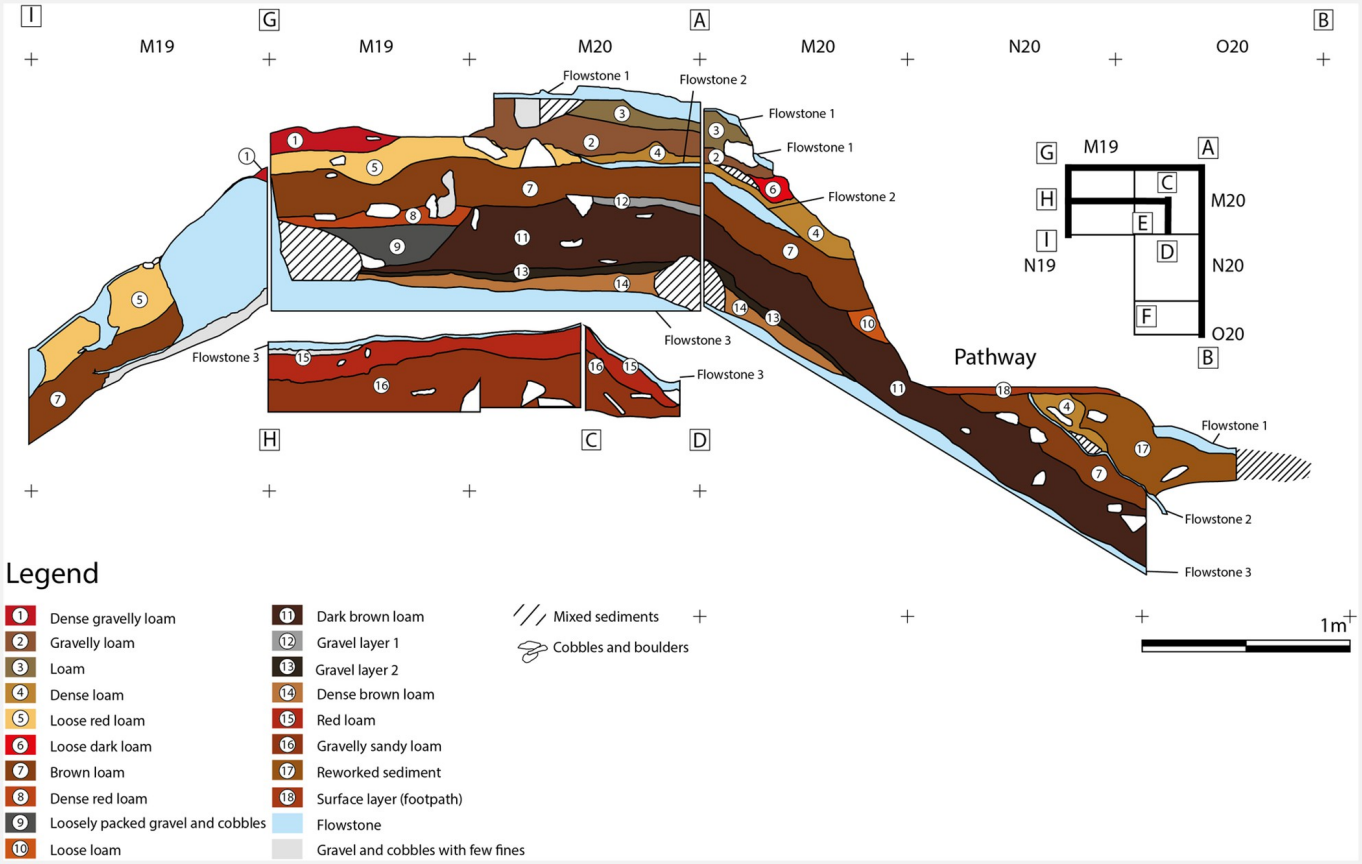
Zone 2 sections.

**Zone 2** is located in the Sala del Saco (sector I.E.) [3], on a spiralling side path that leads laterally from the original entrance across the cone. When the path was built, in the 19^th^ century, it cut through the base of the cone for several metres, creating a section which is over 1 m in height. Here five square metres were cut into the cone and subsequently excavated (Fig 5). Three massive layers of flowstone were found to separate sedimentary deposits (Fig 6). Owing to the destruction of the flowstone layers 1 and 2 in the 19^th^ century and the steep slope, the stratigraphy of zone 2 is highly complex. The two upper flowstones in square M19 and in M20 were destroyed probably during the construction of the path. Only the third flowstone layer, which runs beneath the pathway, was still intact when the excavation began.

Sediment analysis and radiometric dating of the upper part suggest a mixture of Neolithic and late Upper Palaeolithic layers (Table 1). Flowstone layer 3 was also dated by U/Th (Table 2).

**Table 2:** U/Th Datings of flowstone layers in zone 2 and zone 3 of Cueva de Ardales.

Sample-Nr	Labcode	Zone	Layer	Date BP	±	Sample
Ardales 202.14_C1.1 #1	UEVA 1683	3	C1.1	1726	109	Flowstone
Ardales 202.13_C1.1 #1	UEVA 1663	3	C1.1	1941	99	Flowstone
Ardales 202.13_C1.1 #2	UEVA 1664	3	C1.1	2384	81	Flowstone
Ardales 202.13_C1.1 #3	UEVA 1665	3	C1.1	2448	88	Flowstone
CA16_2 E2/F2 Costra 1 upper layer	Mainz	3	Costra E2/F2	9294	180	Flowstone
Ardales 204.13_C1.3 #1	UEVA 1661	3	C1.3	22135	864	Flowstone
Ardales 204.13_C1.3 #2	UEVA 1662	3	C1.3	22210	925	Flowstone
Ardales 205.15_C1.4 #2	UEVA 1660	3	C1.4	33810	768	Flowstone
Ardales 205.15_C1.4 #1	UEVA 1659	3	C1.4	35493	1081	Flowstone
CA16_2 E2/F2 Costra 1 lower layer	Mainz	3	Costra E2/F2	42940	934	Flowstone
CA16_2 Costra 3 Zone 2 upper layer	Mainz	2	Costra 3	8590	100	Flowstone
CA16_2 Costra 3 Zone 2 lower layer	Mainz	2	Costra 3	14700	120	Flowstone

The topmost area of flowstone layer 3 was dated to 8,590 ±100 BP and the lowermost to 14,700 ± 120 BP. Radiocarbon dates from charcoal fragments above the third flowstone range from 3,935 ± 50 calBP, immediately below flowstone layer 1, to 19,268 ± 146 calBP directly above flowstone layer 3. The Holocene dates cluster around 7,000 calBP and 4,000 calBP indicating human activity in the early Neolithic and the late Neolithic/Chalcolithic. Layer 11 yielded a late glacial date as well as several early Neolithic dates, so that disturbances can be assumed. Layers 13 and 14 are well-defined narrow bands containing a small inventory of Upper Palaeolithic artefacts accompanied by a radiocarbon date of 19,268 ± 146 calBP. These layers appear to be intact but the deposition of material from farther up the slope cannot be ruled out. Zone 2 yielded the largest assemblage of stone artefacts in the whole site, with over 500 items (Table 3).

**Table 3:** Lithic assemblages found in zone 2. Layer 1–12 are mixed layers mainly from Holocene context. Layers 13–14 are dated to the Upper Palaeolithic and could represent Solutrean remains. Layers 15–16 were sealed by a flowstone layer and date to the Gravettian.

Layers	Cores	Flakes	Blades	Debitage	Total	Tools
01-dic	2	63	91	356	512	10
13–14	-	13	18	29	60	6
15–16	-	1	-	7	8	-

The mixed layers yielded the highest number of items. Two bladelet cores indicate blank production in zone 2, a notion supported by the presence of six crested blades and five blanks that retain remains of cortex. Several backed bladelets, endscrapers, burins and burin spalls were recorded in the mixed stratigraphic layers. Ceramic fragments from layers 2 and 3 represent Neolithic/Chalcolithic activity.

The number of lithic items, which possibly represent a late Solutrean assemblage, drops significantly in layers 13–14 (Table 3). Diagnostic pieces are lacking. Several backed bladelets, an endscraper and two burins are the only formal tools identified. The layers are only locally present and are absent from the southern sector of M20, where the sample for dating Flowstone 3 was taken.

Below the third flowstone layer, in layers 15 and 16, on a limited surface, a small assemblage of undiagnostic debitage was found (Table 3). This indicates human presence between 24,000 calBP and 28,000 calBP, and probably represents human activity in the cave during the Gravettian.

A number of isolated juvenile and adult human teeth were found in square M19. All teeth come from a disturbed area where Holocene and late Glacial sediments became mixed as a result of construction work and an animal burrow.

Special finds from zone 2 are seven potential ochre pieces (Table 4), chiefly found in the mixed upper contexts, with the exception of one piece found in the Gravettian layer.

**Table 4:** Potential ochre finds from excavation zone 2,3,5.

Chronology	Zone 2	Zone 3	Zone 5
Neolithic	-	3	-
Mixed Neo/UP	6	-	10
Upper Palaeolithic	1	-	15
Middle Palaeolithic	-	37	1
All layers	7	40	26

The faunal remains from zone 2 are dominated by *Oryctolagus*, especially in the mixed layers, while ungulate remains only account for a very small proportion of the assemblage (Table 5). It is only in the Gravettian layers that *Cervus* and *Capra* are more frequent than *Oryctolagus*. Small carnivores as *Vulpes* and *Felis* are present as well. A small number (16) of bone fragments show burn traces. Six of them are identifiable and can be assigned to *Oryctolagus* and *Cervus*. The presence of cut marks is uncertain. Only the burn marks indicate human consumption of animals in the cave.

**Table 5:** Faunal remains (NISP) from excavation zones 2–5 divided by layers.

Zone	Layer	Orcytolagus cuniculus + Lagomorpha	Cervus elaphus	Capra pyrenaica	Capreolus capreolus	Sus scrofa	Equus sp.	Castor fiber	Testudo sp.	Vulpes vulpes	Crocuta sp.	FeIis silvestris	Lynx pardinus	N
2	Layer 1–12 (neolithic + mixed)	117	11	1	-	1	-	-	-	3	-	1	-	**134**
2	Layer 13–14 (Solutrean?)	31	11	-	1	-	-	-	-	-	-	3	-	**46**
2	Layer 15–16 (Gravettian)	2	15	4	-	-	-	-	-	-	-	-	-	**21**
3	1/mixed Neolithic	-	-	-	-	-	-	-	-	-	-	1	-	**1**
3	Layer k-6 (MP)	228	26	11	-	-	-	-	2	2	1	2	-	**272**
4	2/3 Neolithic	20	3	1	-	-	-	-	1		-		1	**26**
5	Layer 1 + 1a (neolithic + mixed)	37	5	-	-	-	-	-	-	-	-	-	-	**42**
5	Layer 2 + 2c + 2d + 2e (Gravettian)	208	54	4	1	-	1	-	2	-	2	-	-	**272**
5	Layer 3 + 3a,b, c,d (Aurignacian?)	27	-	2	-	-	-	-	7	-	-	-	-	**36**
5	Layer 4(MP)	155	14	1	-	1	-	1	-	-	1	-	1	**174**
**N**		**825**	**139**	**24**	**2**	**2**	**1**	**1**	**12**	**5**	**4**	**7**	**2**	**1024**

**Fig 7:**
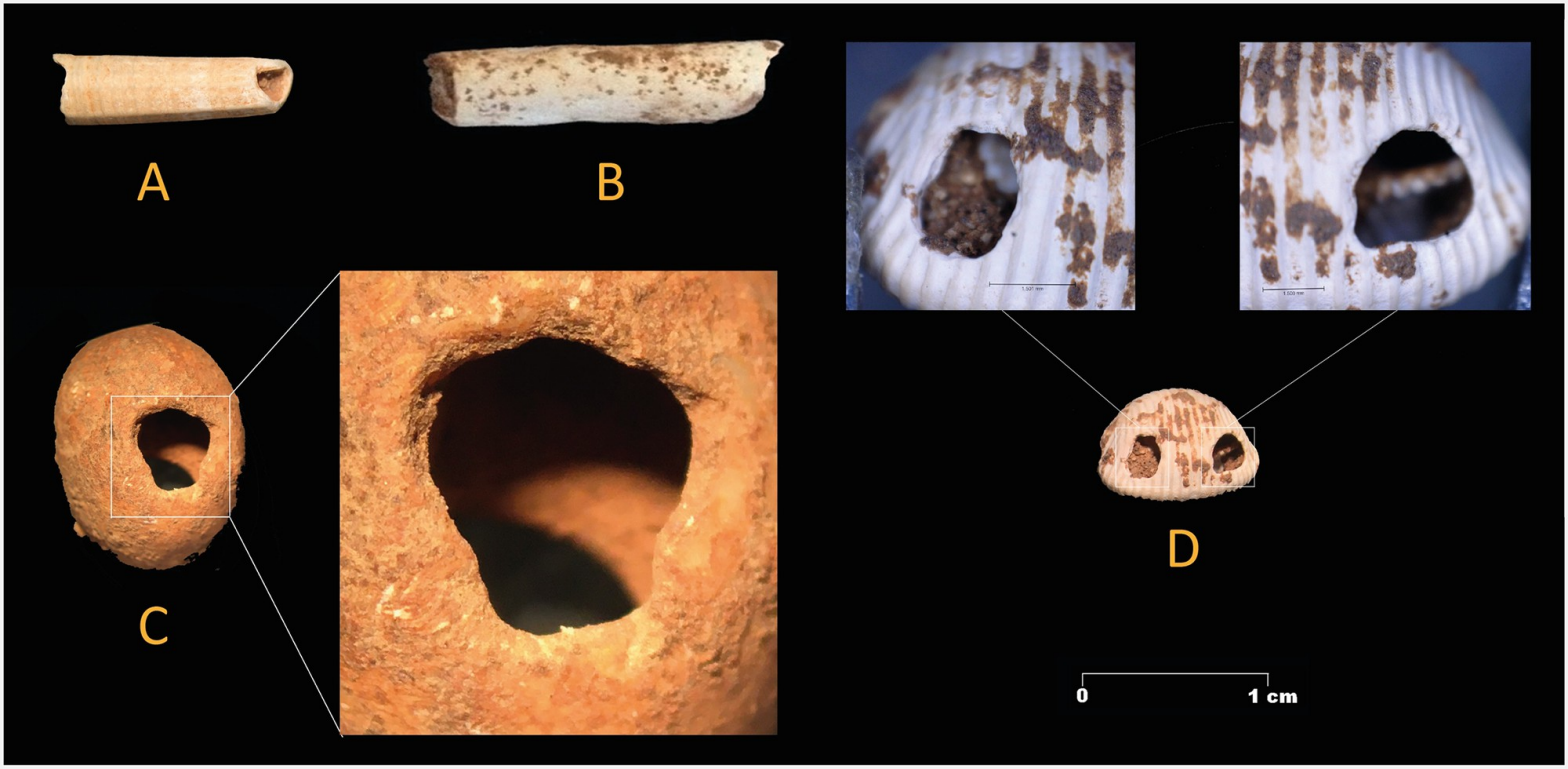
Personal adornment made of mollusks. Left, Gravettian specimens from zone 5, A: Antalis sp.; B: Ditrupa sp.; C: Theodoxus fluviatilis. Right, a double perforated specimen of Trivia monacha found in a disturbed context in zone 2.

Zone 2 also features the presence of five mollusk remains that represent three different species: *Theodoxus fluviatilis*, *Trivia monacha* and *Ditrupa sp*. All of them come from the disturbed sediments in the upper layers of zone 2. Therefore, their context is unclear; they could date from the Solutrean to the Neolithic period. *Theodoxus fluviatilis* (n = 2) occurs in freshwater, while *Trivia monacha* (n = 1) and *Ditrupa sp*., (n = 2), are marine species. All five items show perforations, some of which are natural in origin as in the case of *Ditrupa sp*., while others were made intentionally by pressure or percussion (e.g. *Trivia monacha* and *Theodoxus fluviatilis*). The specimen of *Trivia monacha* displays a double perforation (Fig 7). This indicates that the mollusks were collected and processed to be used as personal adornments. It is possible that those of marine origin were collected *post mortem* on the beach.

Four panels with red signs (I.E.1 –I.E.4) [3] are located near to the excavation area, on the wall to the opposite side of the path. They include patches of different size, fine finger tips and one set of three parallel lines carried out with three fingers.

**Fig 8:**
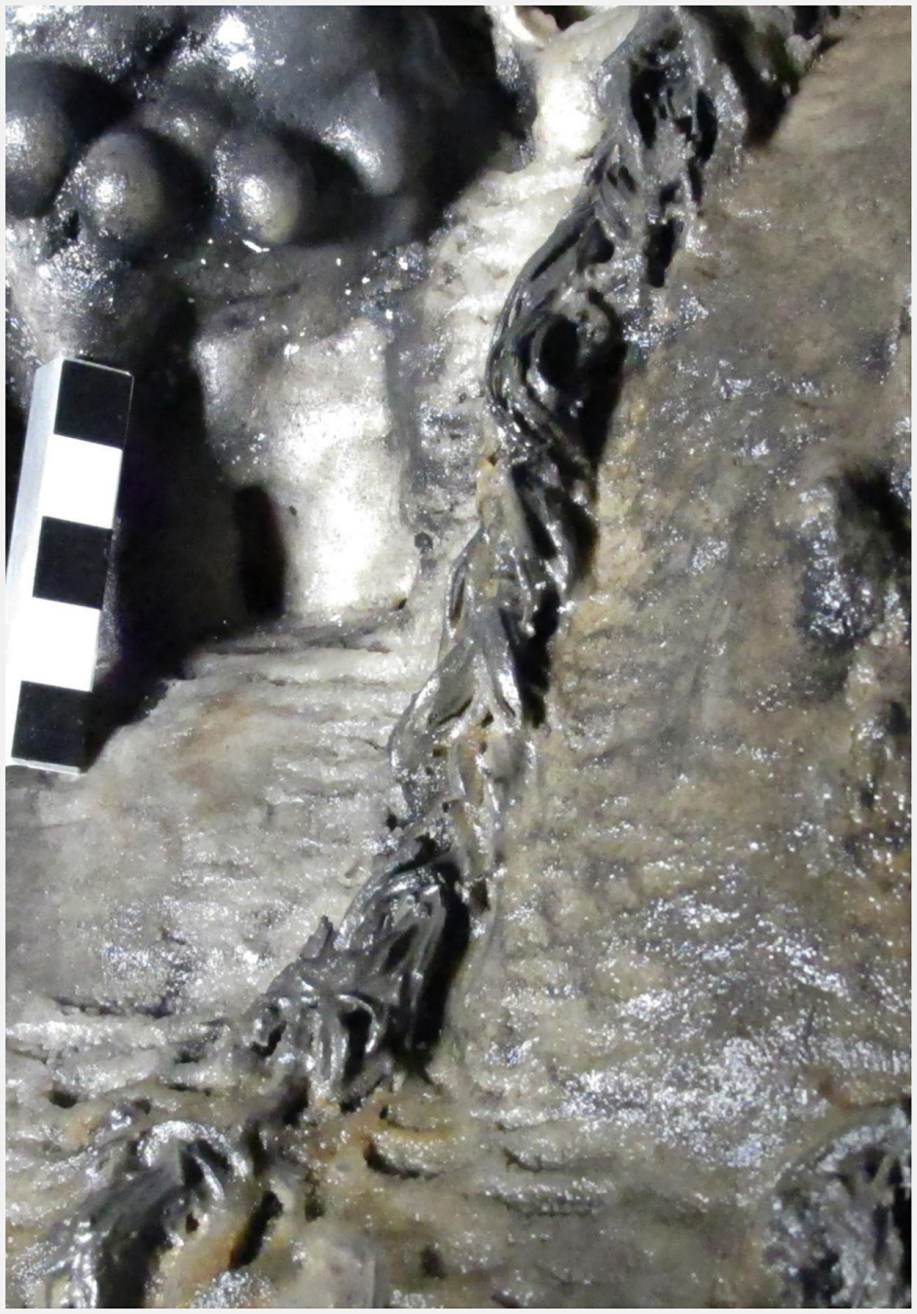
Calcified rope suggesting that the cave was visited in historical times.

**Zone 5** is located at the foot of the sediment cone where the slope gradually flattens out in the Sala de las Estrellas (sector II.A) [3]. In this area the surface flowstone layer is very thin and discontinuous. Ten square meters were excavated (Fig 7). A large charcoal sample was collected from the surface in 2011, around one metre to the south of the excavation area, which was set up in 2015. Its age (347 ± 71 calBP) indicates that the cave was visited by humans in the late 16^th^ or early 17^th^ century. This date matches with that yielded by a fragment of rope (405 ± 70 calBP) (Table 1). The rope was discovered on a rock ledge situated 36 meters away from zone 5, where it was covered and protected by a calcite crust (Fig 8). This bears witness of a human visit to the cave in historical times, and might indicate that access to it was only possible with the help of technical aids. The two dates suggest that the cave must have been, apart from the sealing of the two entrances by sediments, somehow accessible at that time.

**Fig 9:**
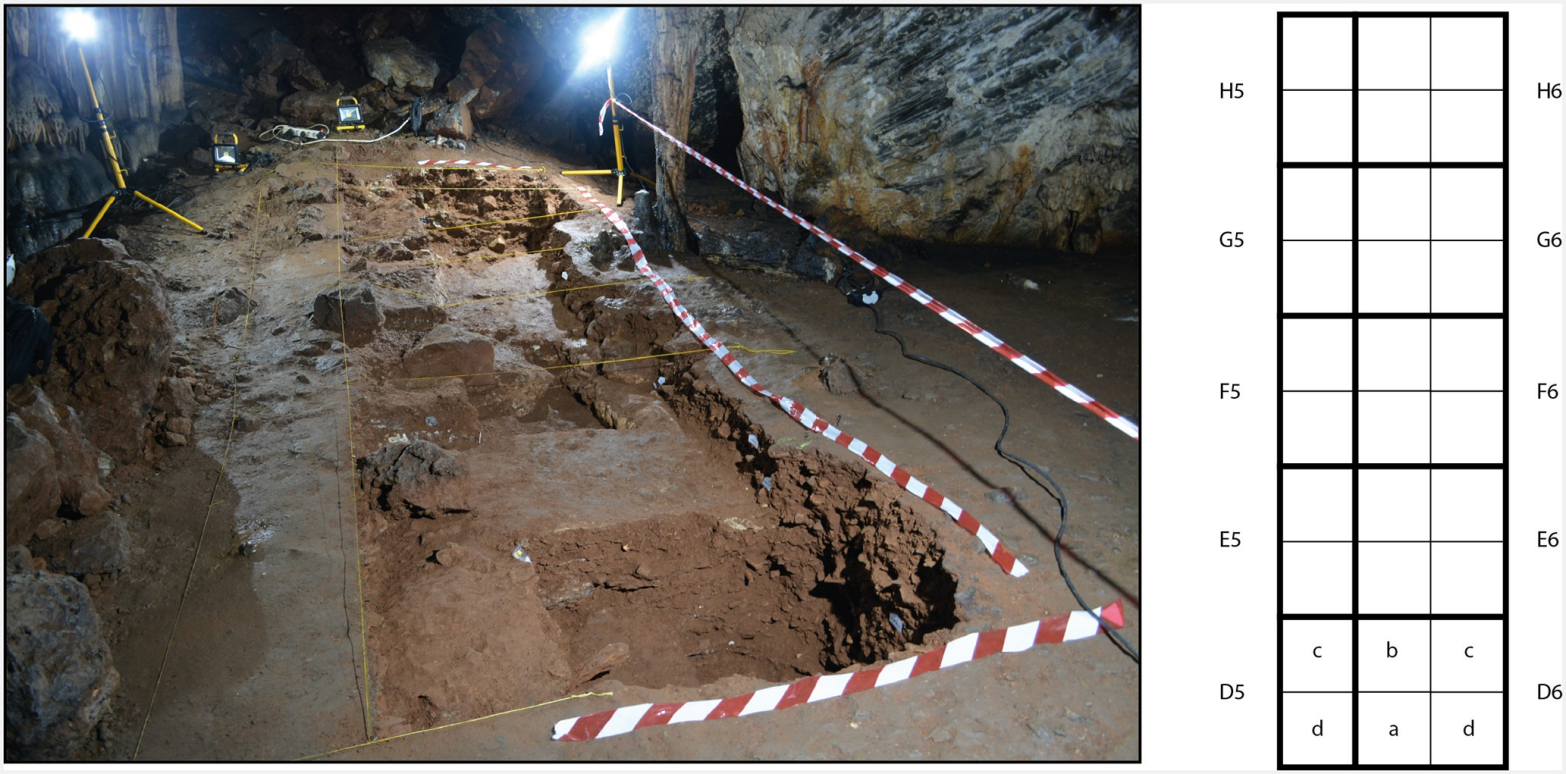
Post-excavation view of zone 5. The plan shows the arrangement of the excavation squares.

**Fig 10:**
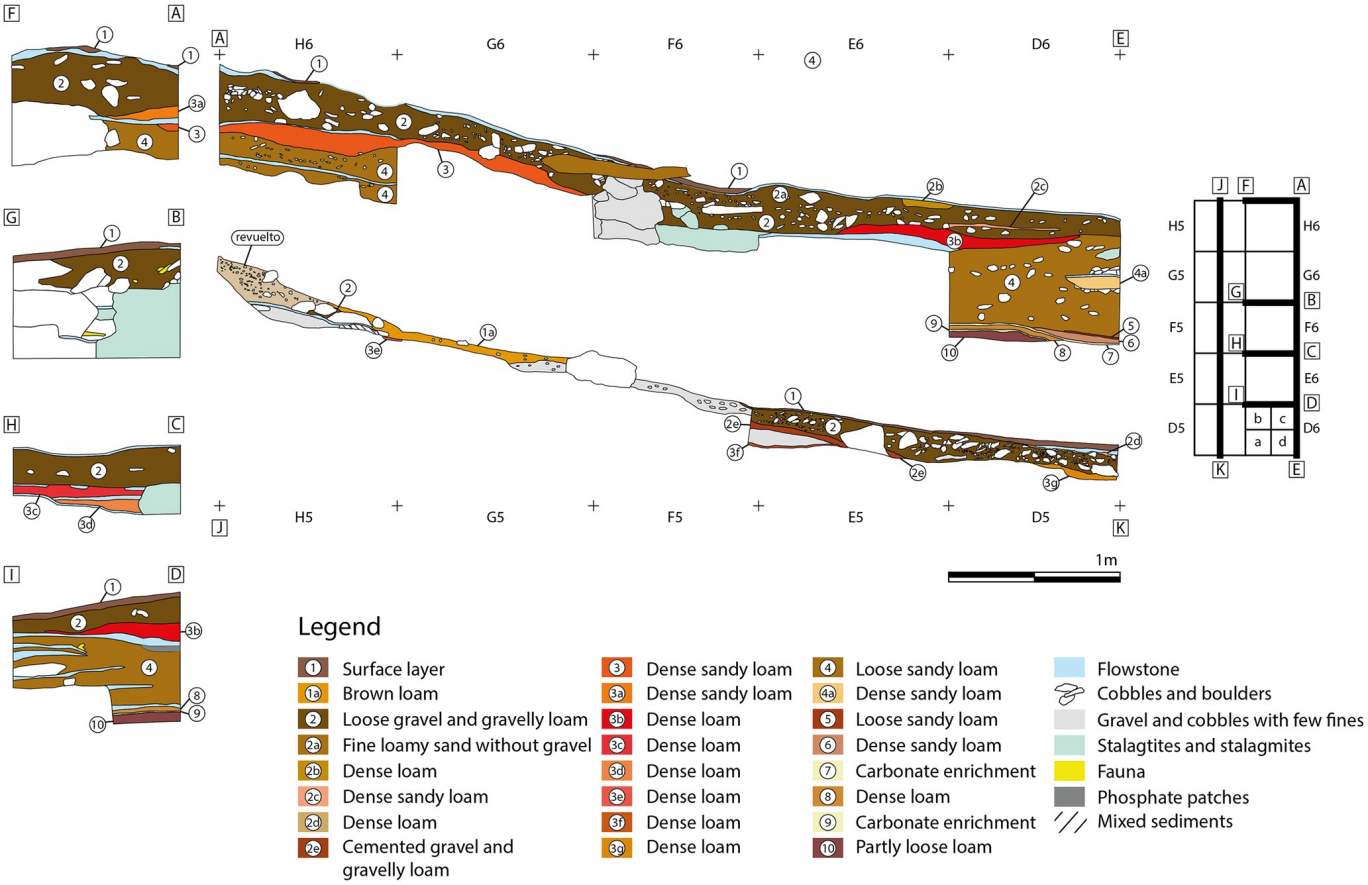
Sections in zone 5.

The sequence in zone 5 consists of fine geological horizons, which are partly intersected by thin layers of flowstone and cemented areas. In addition, fallen speleothems prevent smooth succession of layers in the excavated area. The surface was partially disturbed by trampling or other activities (Fig 9). The sequence (Fig 10) begins with the superficial layer 1. In the eastern part of the trench this layer is separated from the underlying sediments by a thin flowstone layer which is missing in the northwestern part of the excavation area, where the surface of squares H5 and G5 was disturbed. Layer 1a yielded a Neolithic date 7,059 ± 73 calBP and from the disturbed area we obtained an Upper Palaeolithic date 24,505 ± 176 calBP. Underneath lies layer 2, which can be found throughout the trench. Locally, layer 2 displays fine or dense loamy lenses which are named 2a and 2b (in square E6); 2c and 2d (in square D6) and 2e (in E5). In E6, where the flowstone layer had been partly removed, layer 2a yielded a late Neolithic/Chalcolithic date (4,387 ± 74 calBP). Charcoal samples from layer 2 yielded several Upper Palaeolithic dates ranging from 26,421 calBP to 32,564 calBP (Table 1), which suggests human activity during the Gravettian.

A series of compact loamy sediment layers underneath layer 2 were defined as layer 3 (including further subdivisions (3a—3g). In some areas layer 3 was separated from the overlying layer 2 by discontinuous small lenses of flowstone. In other areas this loamy layer appeared above the flowstone. Layer 3a (in H6) was dated to 35,732 ± 343 calBP. A charcoal sample from layer 3b (in D6), found in association with the interface between layer 3 and the underlying layer 4 dates to 35,956 ± 300 calBP (Table 1). These dates are older than the Gravettian occupation.

In H6 and D6 layer 4 lies beneath layer 3 (beneath the flowstone) and layer 3b (above the flowstone). Multiple discontinuous flowstone layers were found to intersect layer 4. Charcoal from between the flowstone layers in square H6 date to 43,402± 553 calBP and 43,619 ± 601 calBP, and a charcoal sample found further down in layer 4 proper, in square D6 dates to 46,374 ± 1,113 calBP. Beneath these Middle Palaeolithic sediments a series of very fine layers (layers 5–10) were identified in square D6, but these were archaeologically sterile, with the exception of some micro faunal remains.

Despite constituting the largest excavation area, the lithic inventory from zone 5 only comprises 84 items, including those with unclear stratigraphic assignment (Table 6).

**Table 6:** Lithic assemblages from zone 5. Surface layer 1 is a mix of Holocene and Gravettian finds. Layer 2 dates to the Gravettian. Layer 3 is slightly earlier than layer 2 and could belong to an Aurignacian context. Layer 4 dates into the Middle Palaeolithic.

Layers	Cores	Flakes	Blades	Debitage	Total	Tools
Surface 1	-	2	3	7	45	2
2	-	3	5	17	25	2
3	-	-	-	2	2	-
4	-	-	1	1	2	-

**Fig 11:**
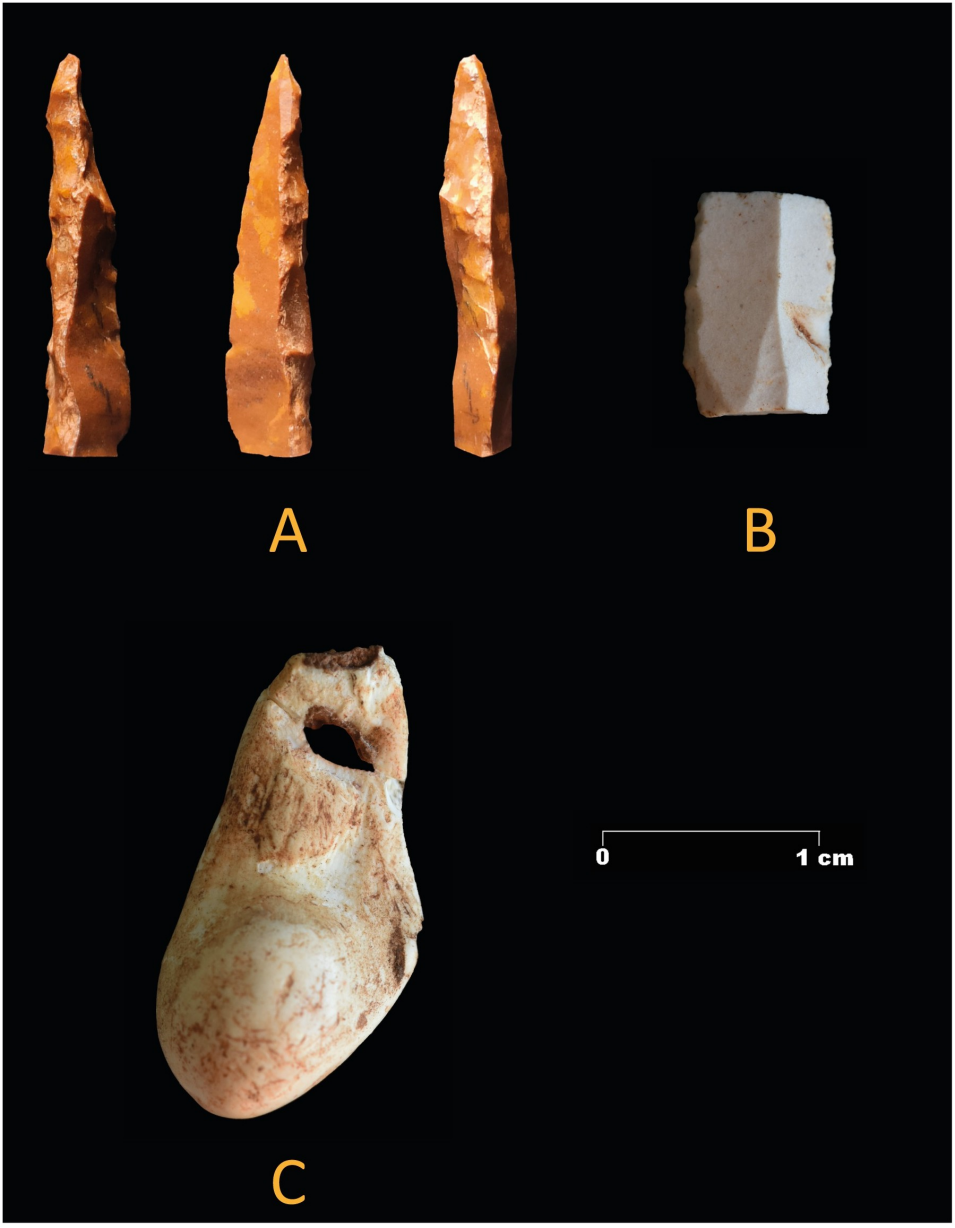
Special finds from layer 2, zone 5. a) broken tip of a Gravette point; b) medial fragment of a marginal inversely retouched bladelet; c) perforated red deer canine.

Most items come from the uppermost layer. No cores are recorded in the whole sequence and none of the lithics present traces of cortex. Two burins from surface layer 1 and two burin spalls from layer 1 and from layer 2/3 are recorded. The tip of a micro point from layer 2 (Fig 11) supports the Gravettian chronology of the layer. The fragment of a retouched bladelet with an inverse marginal retouch similar to Dufour bladelets could indicate an Aurignacian component but would also fit a Gravettian context [11]. Layer 2 also yielded a perforated tooth of *Cervus elaphus* (Fig 9). Other items of special interest are 26 potential pieces of ochre (Table 4). Except for one piece, dated to the Middle Palaeolithic, these pieces come from Upper Palaeolithic or mixed surface layers.

Several isolated human bones and teeth were found on the surface of zone 5. Parallels to this exist in Sala del Saco and in Sala de las Estrellas, where isolated human bones are repeatedly found on the surface, both redeposited and encrusted in the flowstone.

From the surface layer to the Middle Palaeolithic layers faunal remains are dominated by *Oryctolagus* (Table 5). More than 50 remains of *Cervus elaphus* in the Gravettian layer represent the largest group of remains belonging to a single ungulate species in all the excavated areas. These were found alongside single finds of *Capra sp*. *Capreoluscapreolus* and *Equus sp*. The presence of four ungulate species together is unique in the four excavated zones. Interestingly, two specimens of *Crocuta* are present as well. Also of interest is the recording of two turtle remains. Like in zone 2, only eight pieces show traces of burning. Except for one fragment of *Cervus* from layer 2, all burned fragments are undiagnostic. No other evidence for human impact could be established. Interestingly, the excavation of layer 4, which yielded two lithic artefacts, also resulted in the identification of two burnt bone fragments.

The largest assemblage of mollusks (n = 11) in zone 5 comes from layer 2 (Fig 7). Apart from *Theodoxus fluviatilis* (n = 1), *Trivia sp*. (n = 1) and *Ditrupa sp*. (n = 7), already reported in zone 2, another marine species (*Antalis sp*.) is present (n = 2). Both *Antalis sp*. and *Ditrupa sp*. present natural perforations, while *Trivia* sp. and *Theodoxus fluviatilis* show perforations made by simple direct pressure/percussion on their dorsal margin. They were used most likely as personal adornments. This type of decoration is rare in Gravettian contexts in the Mediterranean region [12]. Their occurrence in Cueva de Ardales, over 40 km away from the coast is of particularly interest.

The stratigraphy in zone 5 is complex. Flowstoneformations are thin and discontinuous, unlike in zone 2. The main find in layer 2 is dated to the Gravettian period and some diagnostic finds can likewise be dated to this period. It is followed locally by narrow sediment layers which are slightly earlier in date than the Gravettian layer. Between this and the bedrock there is a thick accumulation of sediments (layer 4) dated to the Middle Palaeolithic.

Zone 5 is framed by two areas which display simple red motifs (Sector II.A.3-4) [3]. At the eastern edge of the trench (Sector II.A.4) [3], several abstract red paintings were found on a speleothem column. One of the paintings yielded a minimum U/Th date of 28,590 BP (ARD 11 A-D; Hoffmann et al. 2018). At the western edge of the excavation lies a huge stalagmite boss (Sector II.A.3) [3] which presents a whole series of non-figurative red marks painted inside the curtains. These motifs yielded a number of U/Th dates (ARD 12–13, ARD 14–15, and ARD 16) [9]: curtain 8 revealed a minimum date of 65,520 BP; curtain 5 a minimum date of 45,940 BP; and a red motif on curtain 5–6 gave a minimum date of 45,290 BP and a maximum date of 48,710 BP. These dates match the radiocarbon dates obtained from the Middle Palaeolithic layers of zone 5.

**Fig 12:**
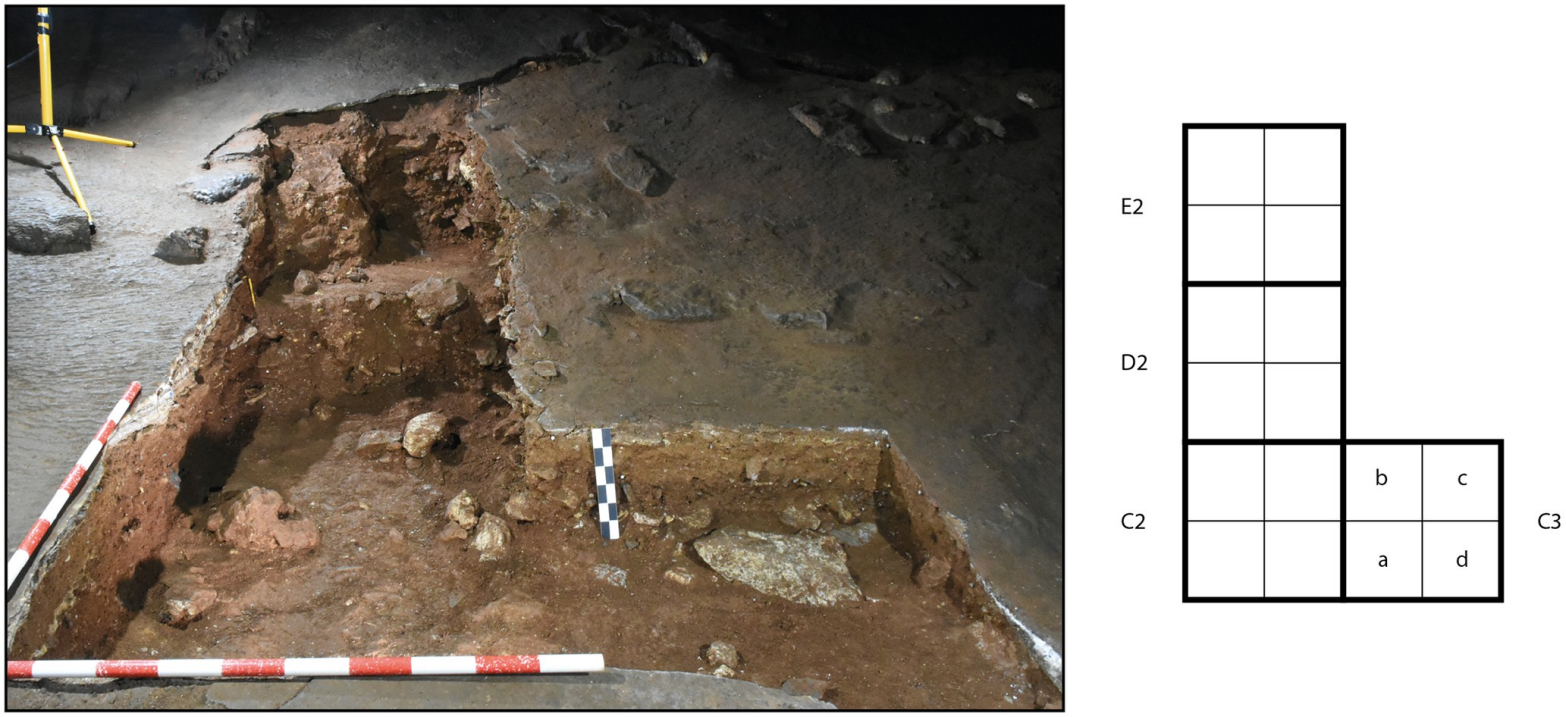
Post-excavation view of zone 3. The plan shows the arrangement of the excavation squares.

**Fig 13:**
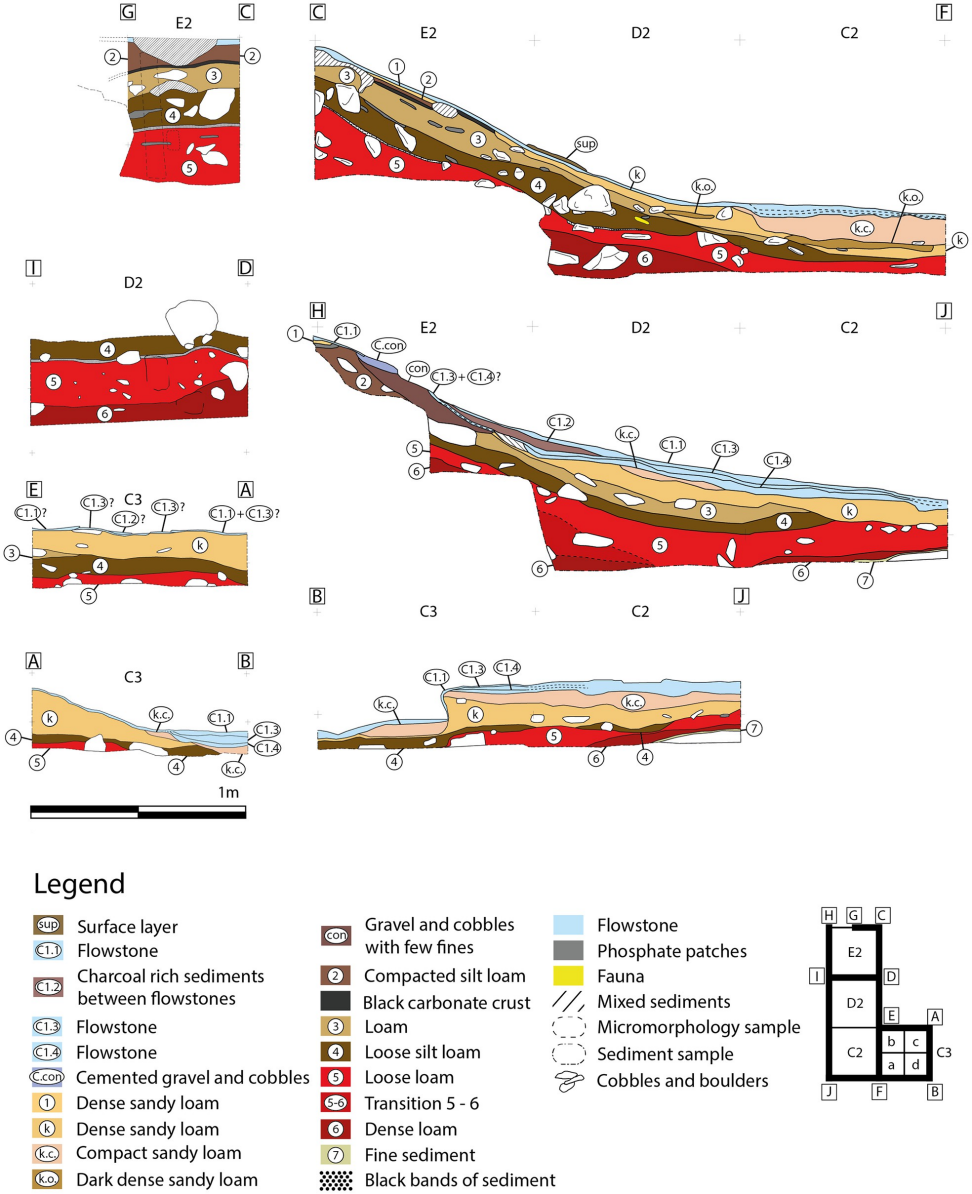
Sections in zone 3.

**Zone 3** is located in the eastern part of the Sala del Saco (Sector I.A) [3] (Fig 12). It is situated in an area where the sediment cone is shallower than in zones 2 and 4 and runs out in front of the cave wall. As this part of Sala del Saco was not disturbed by the construction of the staircase, the superficial flowstone remained largely intact. At a location where it collapsed probably due to trampling the flowstone was further opened for the initial excavation in 2011. In later campaigns, the excavation area was enlarged. The flowstone in zone 3 presents, a variable thickness: it is thicker in the southern and the southwestern sector, than in the northern and the eastern sectors. It comprises at least three different layers, which can be easily traced in squares C2 and C3 (Fig 13).

Top flowstone layer C1.1 sits on top of a sedimentary lens, labelled as C1.2, which is most clearly attested in square C2 and in the western section. This sediment contained large amounts of charcoal. Two further layers of flowstone C1.3 and C1.4, are found beneath. The accumulation of charcoal might suggest the presence of a hearth in a shallow depression on the surfaces of layer C1.3. In the western section (square E2) underneath C1.3 a deposit of gravel and cobbles with few finds, which may be linked to C1.4, was observed.

A series of U/Th samples was taken from C1.1, C1.3, C1.4 and from the topmost flowstone layer in the northern section of E2 (Table 2). In addition, charcoal samples were taken from C1.2 and from a sedimentary lens found in the interface between C1.3 and C1.4 (Table 1). Flowstone layer C1.1 yielded the most recent U/Th date, ranging from 1,700 to 2,400 BP. A charcoal sample from C1.2 dates to ca. 7,000 calBP. Another charcoal sample from the surface, which was found in situ underneath a large rock, could also be related to the same event. Furthermore, a single *Felis silvestris* bone was recovered in the 2011 excavation (square E2) and dated to 6,352 ± 43 calBP. The bone was found within a micromorphology sample from the section. Here, the flowstone layer was already broken before the excavation started and the bone is most likely intrusive. The upper most layer of the flowstone sample from the northern section in E2 is earlier than C1.1. and was dated to 9,294 BP.

Flowstone layer C1.3 is dated to 22,000 BP and C1.4 to 35,000 BP. A charcoal fragment found between two flowstone layers in square D2 dates to ca. 31,500 calBP which perfectly matches the U/Th dates. The bottommost flowstone layer in squares E2/F2 was dated to 42,940 BP. It marks the starting date for the formation of flowstone in zone 3.

Beneath the flowstone sequence, sediments are relatively loose. The upper-most part of these layers, underneath flowstone C1.4, was labelled as layer 1 or layer k. In square E2, this yellow, sandy sediment is visible as a very discontinuous thin layer. As the connection to square D2 could not be clearly established in later excavation campaigns, this sediment which sits directly beneath the flowstone sequence in the other squares was labelled as “layer k”, although it is highly likely that “k” and “1” are the same sedimentological unit. Layer 1/k is fairly thin in the northern sector of the excavation area, and becomes thicker further south. In squares C2, C3 and some areas of D2 along the western section, k appears to be strongly cemented with the flowstone located above. In the eastern section, two darker inclusions were observed, although their texture is similar to that of layer k.

A charcoal sample from layer k in square D2 is dated to 52,950 ± 1,710 calBP suggesting that the sediments directly below the flowstone sequence are already of Middle Palaeolithic age. Layer 2 was only identified in square E2, and near the eastern section only forms a very thin layer. No finds could be clearly associated with this layer. The sediment is a relatively compact brown silt loam. Layer 3 is mainly visible in the northern squares. This layer is fine and slightly clayey, and in square E2 it features yellow phosphorus patches. Here charcoal samples could only be taken during the 2011 campaign. A new layering system was established in 2016 and it is most likely that the two charcoal samples dated to 54,815 ± 2,708 calBP and 55,986 ± 3,033 calBP are related to layer 3.

Layer 4 lies beneath layer 3 is constituted by finer and looser sediments, and is thicker in the northern sector of the excavation area, disappearing in square C2. Only one radiocarbon date was taken from this layer (square E2), which dates to >58,000 BP.

Layers 4 and 5 are separated by a fine layer of blackish sediment, which is especially prominent in squares E2 and D2, and less visible in the remaining squares. Layer 5 is loosely packed, reddish and more clayey than layer 4. It is relatively thick in all squares, and thickest in the northern sector of the excavation area. The only radiocarbon date obtained from this layer also yielded an age >58,000 ka BP (square E2).

Layer 6 is made up of reddish, loam and more compacted than layer 5. These sediments are visible in D2, the western sector of E2, and in C2. No radiometric dates have been obtained from this layer. Finally, the bottommost layer 7 was only identified in C3 directly above a large rock.

**Fig 14:**
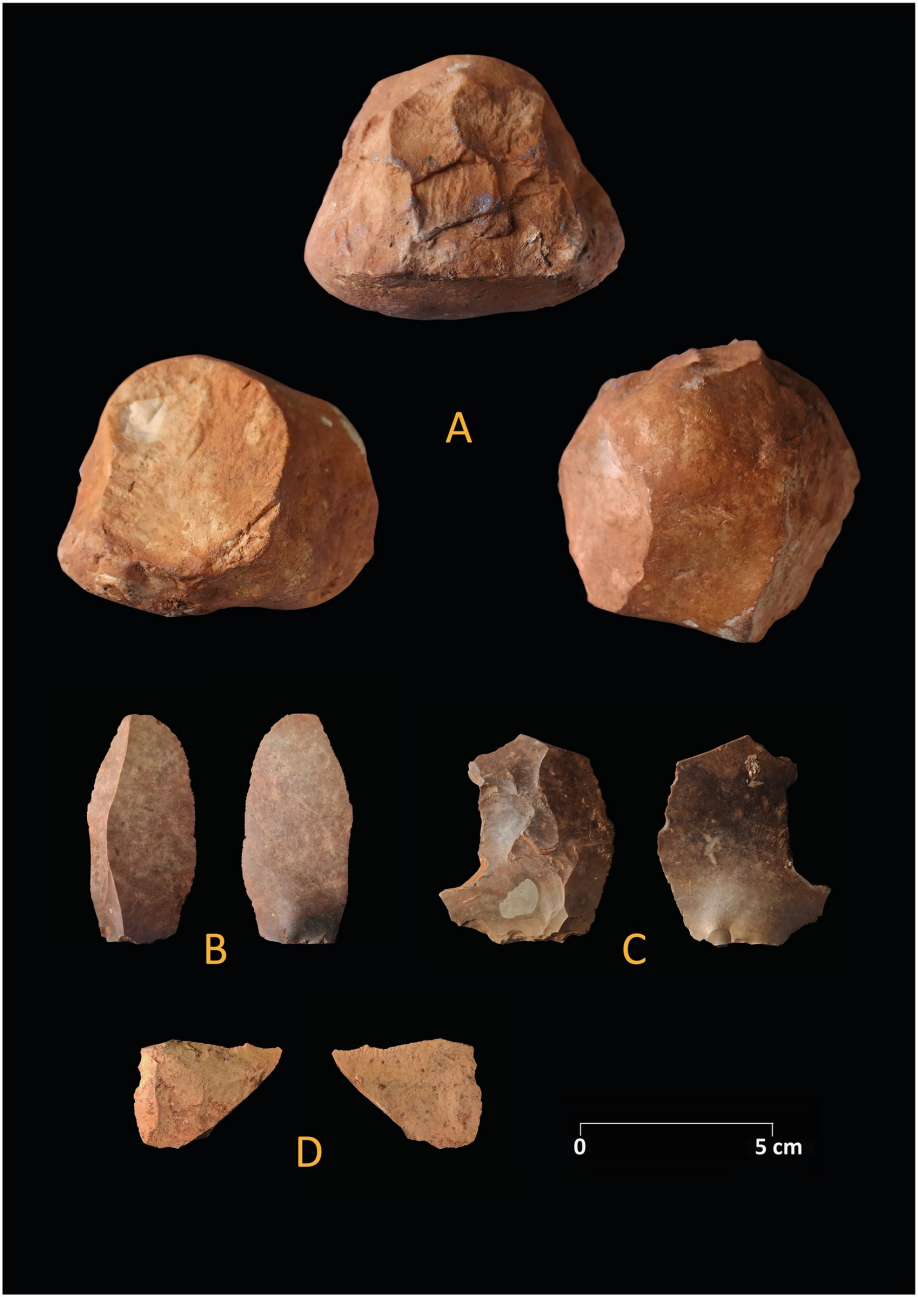
Lithics from the Middle Paleolithic layers of zone 3. A: Quartzite core or heavy duty tool, B: Blade, C: Levallois flake, D: Sidescraper.

The lithic inventory of zone 3 (Table 7) is small and formal tools are rare (Fig 14). In total 154 items were recorded and 120 pieces were securely assigned to a layer; 98 pieces represent small debitage. A backed bladelet was found in the mixed surface layer. One sidescraper with marginal transversal retouch was found in association to layer k or layer 3 while extracting a big stone and a denticulate was found in layer 5. Several flakes reveal a reduction through the Levallois concept. A large object from layer 5 could be a heavy duty tool or a very simple quartzite flake core. Interestingly the density of pieces in the Middle Palaeolithic inventories increases with depth.

**Table 7:** Lithic assemblage from zone 3. Finds from the surface layer date mainly to the Holocene. Layers k to 6 all date to the Middle Palaeolithic.

Layers	Cores	Flakes	Blades	Debitage	Total	Tools
Surface	-	-	2	3	5	1
k	-	-	1	3	3	-
3	-	3	-	8	11	-
4	-	-	-	11	11	-
5	1?	6	3	25	36	1
6	-	4	2	48	54	-

A total of 40 potential ochre pieces were found in zone 3, which is by far the largest sample of ochre in the whole site (Table 4). Two pieces that were enclosed in the upper flowstone layers date to the Neolithic; one piece comes from the mixed surface layer, and 37 were found in association with the Middle Palaeolithic layers. They are distributed quite evenly across all layers, except for a discrete cluster of thirteen pieces found in layer 5.

The faunal remains from zone 3 (Table 5) are dominated by rabbit. The number of red deer remains is small. Several turtle remains were identified as well. Interestingly three carnivore species are documented from the Middle Palaeolithic levels, namely hyena and two small species (wild cat and red fox).

**Fig 15:**
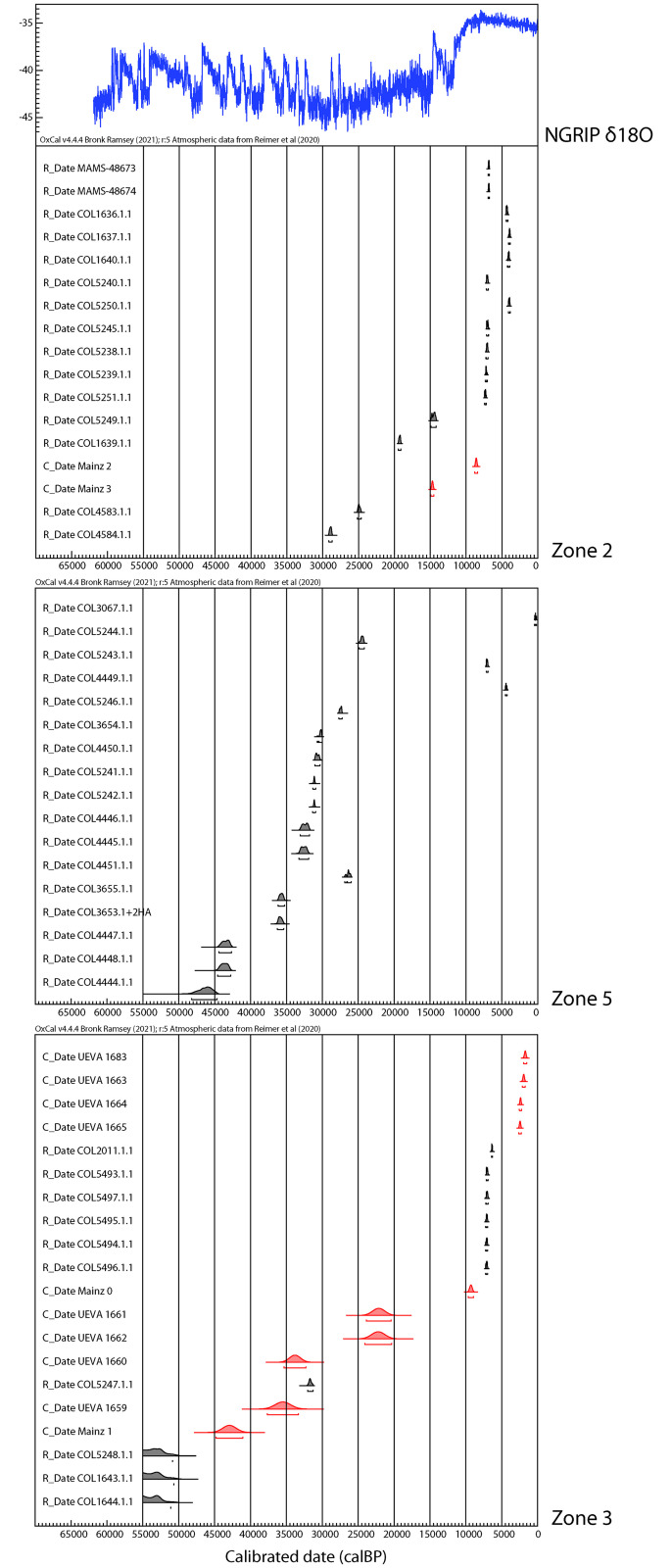
Radiocarbon dates for the Palaeolithic occupation of Cueva de Ardales.

Zone 3 was completely covered by a thick layer of flowstone. Its formation started around 43.000 BP at the end of the Middle Palaeolithic and grew constantly thereafter; it is at its thickest in square C1. It was locally removed only in the northern section of square E 2, probably by being repeated walked on in historic or prehistoric times. The evidence collected in zone 3 mostly documents the Middle Palaeolithic (or earlier) uses of the cave, as sediments directly underneath the flowstone date to 50,000 calBP or are even earlier (Fig 15). Later dates, however, are recorded from the flowstone sequence itself, dating to historical, Neolithic as well as the Upper Palaeolithic periods. Only layer C1.2 included archaeological material that could be dated to the early Neolithic. The Middle Palaeolithic sequence yielded small lithic assemblages, faunal remains and an important number of potential pigment lumps. The latter are of special interest because directly behind and above the excavation zone where the Fe-rich materials were found there is a huge decorated panel featuring more than 200 red dots painted with one, two or three finger tips (sector I.A.11) [3].

## Discussion

When the excavations in Cueva de Ardales were planned, it was clear from the outset that only a small area of the cave could be investigated in the first phase. We decided to excavate the entrance because it presented large sectors that were sealed by thick flowstone formations that protected the sediments underneath from later intrusions. The archaeological windows opened during the different excavation campaigns cannot cover all aspects of the prehistoric occupations of the cave, but they are significant enough for a first interpretation of the site.

The dates yielded by the excavated areas present a clear sequence of human occupations in the Cueva de Ardales (Tables 1 and 2, Figs 13 and 15). According to the evidence collected in zone 3, humans first entered the cave more than 58,000 years ago during the Middle Palaeolithic. In this zone, the occupation ends around 43,000 BP at the latest, but most likely around 50,000 BP, with the formation of the flowstone that sealed the Middle Palaeolithic sediments. Layers 6-k yielded small assemblages of stone artefacts with Middle Palaeolithic technology, faunal remains and numerous lumps of potential ochre. Apart from the human presence, we documented the presence of large carnivores such as hyena. The lithic inventories and faunal remains are not informative in terms of human activities. In parallel to the end of the Middle Palaeolithic occupation of zone 3, the presence of human activity in the lower sedimentary package in zone 5 was detected. Charcoals from layer 4 indicate an age between 43,000 calBP and 46,000 calBP for the upper part of the layer. The underlying layers 5 to 10 were not dated in this zone because, except from the presence of some micro faunal fragments, it appears to be archaeologically sterile.

The nature of Middle Palaeolithic human activity in Cueva de Ardales is difficult to assess. The lithic material clearly shows Middle Palaeolithic features, but is of little significance beyond that. There are no indications of extensive blank production. Fireplaces are lacking and only two undetermined faunal remains, from layer 4 in zone 5, show traces of burning. As the faunal remains do not give any indication of extensive domestic activities, it must be assumed that the four documented carnivore species were most likely responsible for the accumulation most of the faunal assemblage especially if we take into account that the presence of hyena has been attested in the Middle Palaeolithic levels in both zones.

Concerning the 36 potential ochre fragments, the chronology of the layers from which these materials come from agrees with the Middle Palaeolithic dates obtained for some of the red abstract depictions from panel II.A.3 (ARD 14–15: 45,290 BP and ARD 12–13: 65,520 BP) [9]. The panel is located near the western section of zone 5. The youngest date (ARD 11 D:28,590 BP) [9] was obtained from one of the red spots in the series documented on the stalagmite located near the eastern section of zone 5 (panel II.A.4) [3]. Since this is a minimum date, the application of the red pigment could have occurred in the Middle Paleolithic as well, but may date later to the Early Upper Palaeolithic.

Further away from zone 5 in a side gallery, the age of red pigments in a drapery that was broken (panel III.C.3.-2., Cantalejo et al., 2006 a) was dated by U/Th as follows: ARD 26 B: minimum date of 38,640 BP; ARD 28: maximum date of 45,540 BP [9]. Taking into account that in zone 5 the radiocarbon dates of layer 3 (35,956 calBP) points out to an unclear Aurignacian occupation, the hypothesis that the application of the pigment occurred during the Middle Palaeolithic cannot be ruled out.

After the Middle Palaeolithic activity there is a clear hiatus in the stratigraphic sequence. While zone 3 was sealed by the formation of the flowstone, sedimentation continued in zone 5 with layer settling above the Middle Palaeolithic sequence. Two dates yielded by charcoal fragments (35,732 calBP and 35,956 calBP) suggest a gap of over 7,000 years. From a strictly chronological point of view, layer 3 could represent a late Aurignacian occupation of the cave. However, the human presence in the south of the Iberian Peninsula during the Aurignacian is highly disputed. The recently published old excavation material from Bajondillo Cave [13], pushes back the beginning of the Aurignacian by several thousands of years to about 43 ka calBP [14]. This interpretation was however contested [15, 16], so that evidence for an early Aurignacian period in Andalusia before 40 ka calBP still is a matter of debate. In general, the number of sites that can be attributed with some certainty to the Aurignacian techno-complex in Andalusia is extremely small. Besides the Aurignacian layers in Bajondillo, the occupation of Boquete de Zafarraya [17, 18] and level IV in Gorham’s Cave [19, 20] are disputed, and the evidence is unclear. Leaving the regional perspective aside and considering locations south of the 40^th^ parallel north, only a handful of sites can be assigned to the Aurignacian and, in several of these instances, this attribution is open to question. Pego do Diabo [21], Finca Doña Martina (Layer 8 undated), La Boja, Mallaetes, Los Cendres and Ratlla del Bubo [22] are dated to the later stages of the Aurignacian. Recently published data from Lapa do Picareiro present evidence for an Early Aurignacian, predating 40 calBP [23]. Although a good candidate for an Early Aurignacian, the earliest radiocarbon dates come from slightly below the small find scatter of about 40 lithics that were assigned to the basal Aurignacian level, and therefore, they might not be dating the actual occupation of the site.

Even after two decades of intensive research evidence for Aurignacian occupations in Southern Iberia remains scarce [24, 25]. The possibility has been floated that some of these sites might reflect a special variant of early Gravettian [15, 26]. In addition to this, the unclear situation of the Aurignacian in the South of the Iberian Peninsula and the extremely poor data yielded by Cueva de Ardales compels us to review the evidence for Aurignacian occupation in layer 3 with extreme caution.

The human presence in the cave during the Gravettian was documented in zones 2 and 5. In zone 2, this is only based on two radiocarbon dates on charcoal, as the few lithic artifacts found in layers 15 and 16 are undiagnostic. In zone 5, besides two radiocarbon dates, a number of diagnostic finds support the Gravettian chronology of layer 2.

Occupation of the cave in the Solutrean was attested in zone 2. A number of artifacts was documented in layers 14 and 13, right above flowstone layer 3. Based on a radiocarbon date, the layers could be dated to the very end of the Solutrean. However, diagnostic artifacts are lacking. In addition the Solutrean attribution is also open to question because the whole sedimentary sequence above flowstone layer 3 is likely to be disturbed. Even more uncertain is the presence of human activity during the Magdalenian. The second late glacial date on charcoal of (14,485 calBP), comes from a layer of sediments in zone 2 that contains ceramics and has also yielded dates in the early Neolithic.

**Fig 16:**
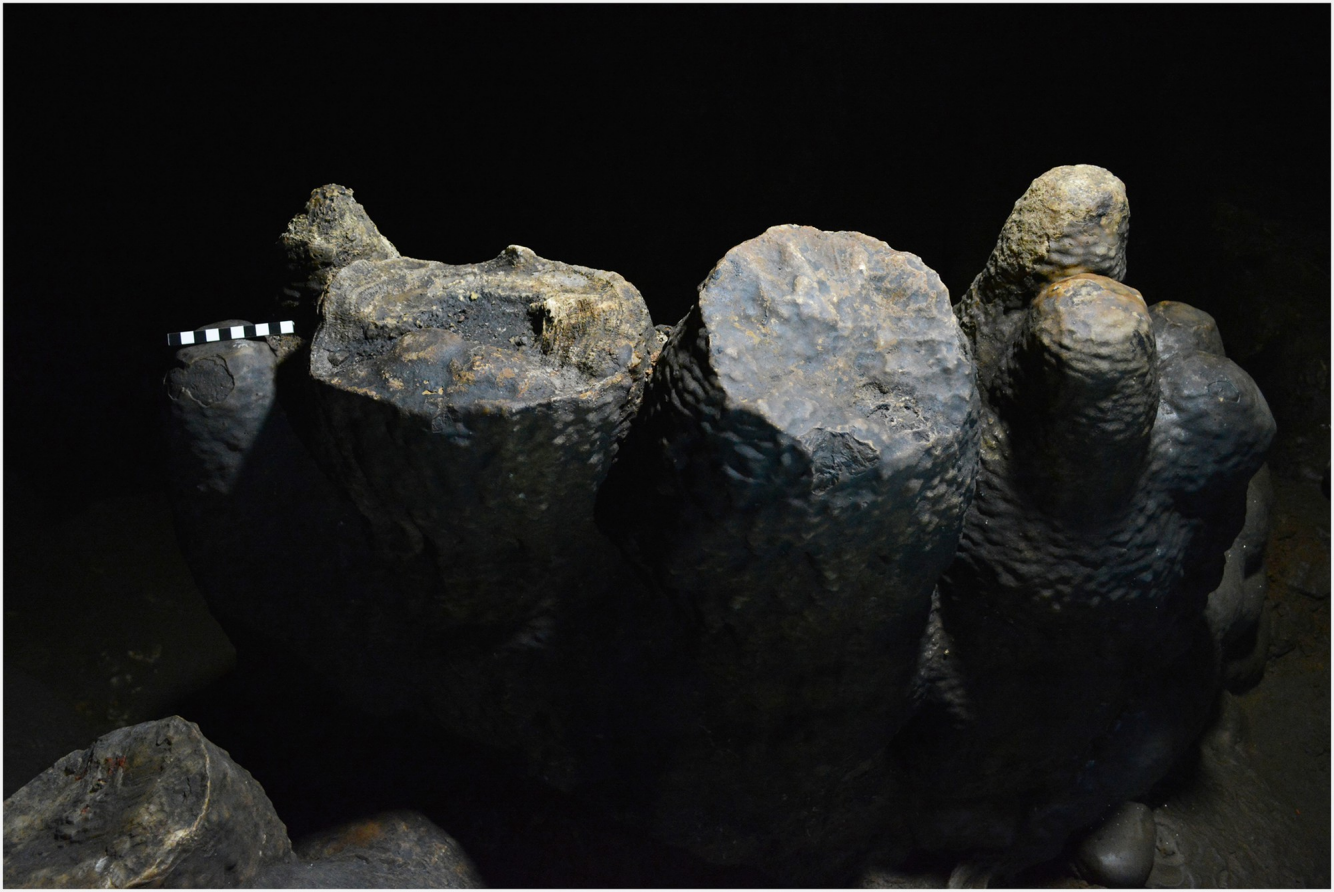
Capped stalagmite used as a stationary lamp during the Epipalaeolithic.

**Fig 17:**
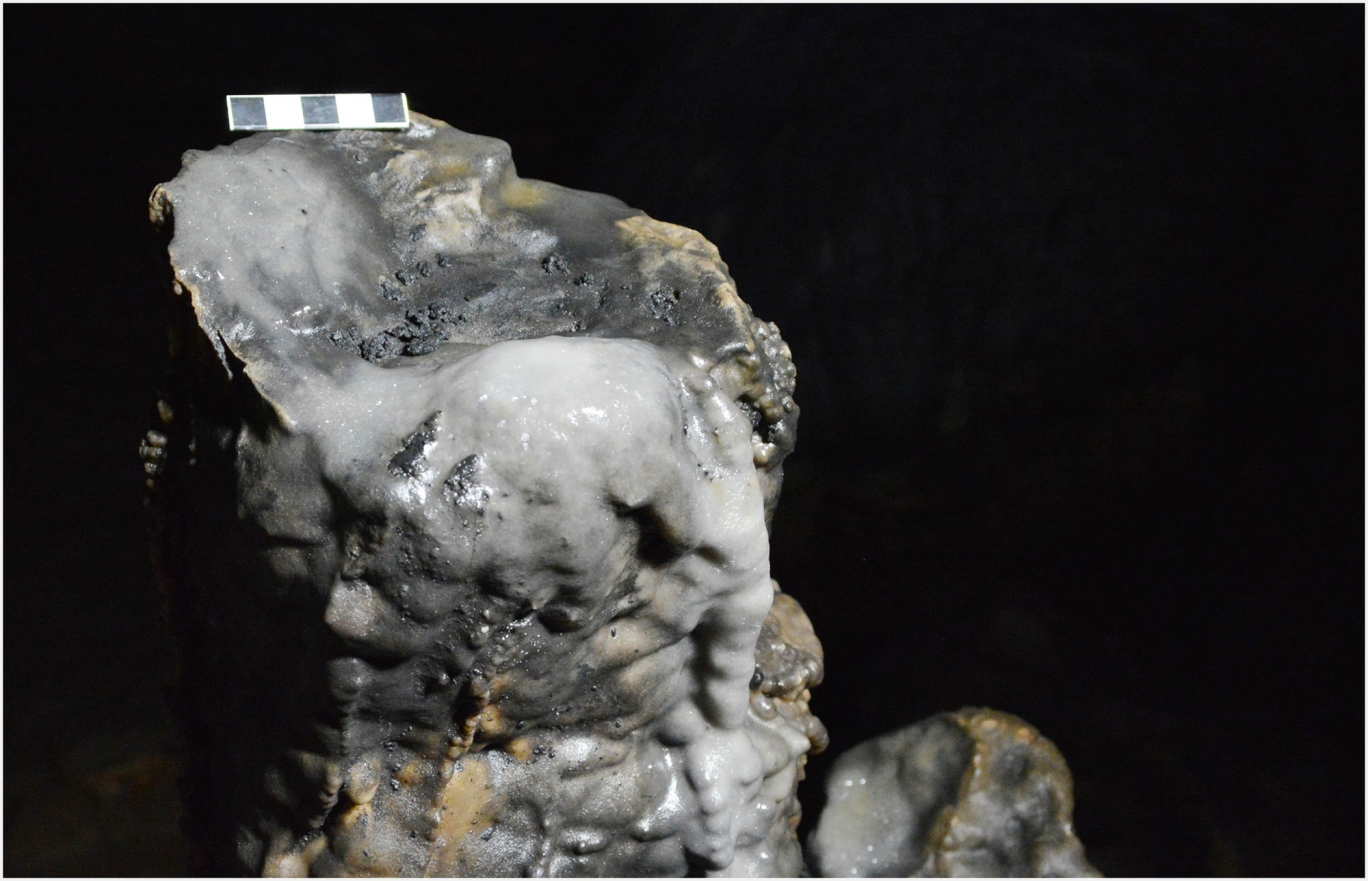
Capped stalagmite used as a stationary lamp during the Neolithic/Copper Age.

An early Holocene date of 11,311 calBP was obtained from a piece of charcoal found at the top of a stalagmite in the Sala de las Estrellas, at a distance of approximately 36 m from zone 5. The stalagmite was capped and slightly hollowed out by percussion at a height of about 100 cm (Fig 16). The presence of charcoal in the resulting depression as well as other fine black residues, indicates that the stalagmite was probably used as stationary lamp. A second stationary lamp, 55 cm tall, also featuring charcoal fragments and other black residues, was found only 3 m away. In this instance, it is thought that the lamp was last used in the Late Neolithic/Copper Age since the charcoal sample yielded a date of 4,664 calBP (Fig 17). It is worth noting that a study of lighting conditions by ray tracing suggests that the cave was essentially closed to daylight during prehistory [27]. So artificial lighting would have been required to circulate inside, by fire, torches or lamps. In this sense, several other capped stalagmites were found in the cave. Some of them were probably used as stationary lamps more recently, but we are certain that at least two of them were used for this purpose in prehistoric times.

Interestingly, the radiocarbon record from the topmost layers in zone 2 suggests a hiatus of approximately 7,000 years. With the evidence available, a significant Magdalenian and Epipalaeolithic occupation of the cave is difficult to establish with certainty. Substantial evidence for the human presence is found in a series of radiocarbon dates from the early Neolithic. Although ceramics and lithics are rare, several human remains found in zone 2 as well as the ones found in the niches located throughout the main staircase, suggest that the cave was used as a burial place during the period. Several pieces of potential ochre were found in Neolithic levels as well. The latest prehistoric activity attested in the cave dates to the transition from the late Neolithic to the Copper Age.

The excavations of the area of Galerías Bajas in the Cueva de Ardales have not found evidence for prehistoric domestic activity that could suggest the use of the cave as a long-term campsite. The traces of human activity are ephemeral and point out to very specific activities related to the symbolic use of the cave. For instance, burial during the Neolithic. The presence of a substantial number of potential ochre lumps is important in this regard. Ochre lumps were documented in all chronological phases, peaking significantly during the Middle Palaeolithic. This supports the idea that the cave was mainly used as a location for rock art from the Palaeolithic onwards. It seems reasonable to assume that the associated campsite was located outside the cave.

In this sense, coring in the parking area in front of the cave revealed disturbed sediments and modern backfill, which suggests that the forecourt of the cave has been heavily altered in the recent past. Nonetheless, the entrance is still preserved in its original form: a hole leading, vertically, into the underground karst system. Access to the cave was only possible by means of a steep sediment cone located right behind the entrance. The excavation of the cone (zone 2) yielded the largest amount of stone artefacts of all excavated areas. This is probably because domestic activities took place directly in front of the cave. This said, there is an open-air site known as Cucarra, a flat area (360 m^2)^ where more than 400 lithic artefacts were recovered from the surface, located barely 100 m downslope from the cave entrance. The vast majority of these artefacts were attributed to the Middle Palaeolithic. Furthermore, small numbers of Upper Palaeolithic or Neolithic blades and retouched blades were recorded in Cucarra. About 100 metres further to the side, at the foot of the slope where the cave entrance lies, there is a karst spring so plentiful that it is used for agricultural irrigation today. This gives further support to the idea, that the main settlement site was probably located in the area outside of the cave and that the cave itself was only entered for symbolic activities. This kind of setting has already been documented in numerous caves featuring Palaeolithic rock art [28, 29].

**Fig 18:**
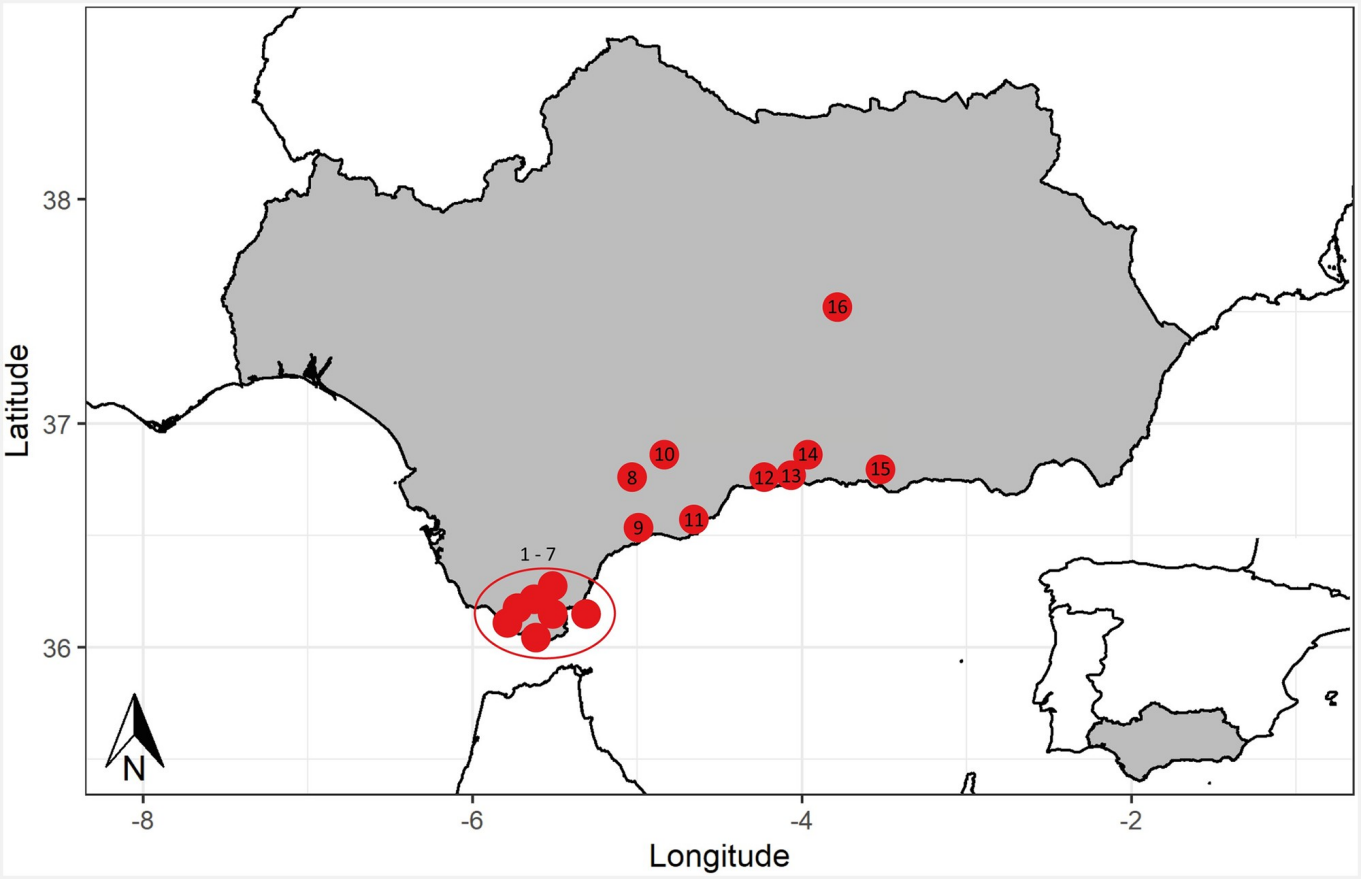
Distribution of rock art sites with non-figurative red paintings and handstencils in Andalusia and Gibraltar: 1: Moro, 2: Estrellas, 3: Palomas I, 4: Palomas IV, 5: Atlanterra, 6: Horadada, 7: Gorham, 8: Pileta, 9, Pecho Redondo, 10: Ardales, 11: Calamorro, 12: Navarro, 13: Victoria, 14: Higuerón, 15: Nerja, 16: Malalmuerzo.

In relation to the regional context of Palaeolithic art in Andalusia and Gibraltar, Cueva de Ardales belongs to a series of 32 sites with Palaeolithic rock art. Half of these host abstract red marks such as dots, finger tips and hand-stencils usually painted with the hands/fingers or by splattering (Table 8; Fig 18). Radiometric dating of rock art in these sites is extremely rare as is generally the case with Palaeolithic rock art. These panels and motives, are broadly dated, based on stylistic criteria, to pre-Solutrean chronologies [30, 31].

**Table 8:** Rock art sites with non-figurative red paintings and handstencils (P = Positive; N = Negative) in Andalusia and Gibraltar.

Site	Province	Type	Chronology	Dots	Finger-tips	Hand stencils	References
Moro	Cadiz	Rockshelter	SOL-MAG		+		[38]
Estrellas	Cadiz	Rockshelter	GRA-SOL	+	+	N	[39, 40]
Palomas I	Cadiz	Rockshelter	GRA_SOL	+	+		[41]
Palomas IV	Cadiz	Rockshelter	GRA-SOL	+	+	N	[42, 43]
Atlanterra	Cadiz	Rockshelter	SOL-MAG		+		[44]
Horadada	Cadiz	Rockshelter	SOL-MAG		+		[44]
Gorham	Gibraltar	Cave	SOL-MAG			N	[45]
Pileta	Malaga	Cave	GRA-MAG	+	+	P	[46, 47]
Pecho Redondo	Malaga	Cave	GRA	+	+		[48]
Ardales	Malaga	Cave	MP-MAG	+	+	P/N	[3]
Toro/Calamorro	Malaga	Cave	GRA	+	+		[49]
Navarro	Malaga	Cave	GRA-SOL	+	+		[50]
Victoria	Malaga	Cave	GRA-SOL	+	+	P	[30]
Higuerón	Malaga	Cave	GRA-SOL	+	+	P	[30]
Nerja	Malaga	Cave	GRA-SOL	+	+		[51]
Malalmuerzo	Granada	Cave	GRA-MAG	+	+		[52]

In Cueva de Ardales, the full scanning of the artistic repertoire [3] revealed that most abstract red motifs are located in the entrance area and the adjacent zones rather than at the back areas. Painted and engraved animal depictions are, on the contrary, dominating the interior areas. This spatial distribution, as well as four instances in which black pigments were laid over earlier red suggests the non-figurative red motifs are the earliest representations in the cave. The U-series dating of calcite accretions superposing some of these non-figurative paintings indicate that they were made at least 65 ka ago [9–32]. In addition, the repeated application of pigment on panel II.A.3 has been attested through pigment analysis, indicating a symbolic use of both, the paintings and the stalagmitic dome harbouring them over more than 20.000 years [32]. In addition, excavations resulted in the discovery of a significant number of potential ochre lumps in all chronological phases, including Middle Palaeolithic stratigraphic units. All this evidences supports the hypothesis that non-figurative paintings represent, indeed, the beginning of a long rock art tradition in Cueva de Ardales.

From a regional perspective, it is worth considering the chronological analysis carried out at Nerja, the well-known cave art site located near the coast, 100 km east of Ardales [33, 34]. There motif distribution and application techniques are fairly similar to those found in Cueva de Ardales. The U/Th dating of calcite accretion covering red motifs (sample GN12-25) yielded a minimum age of 55,848 BP, which matches the results obtained from panel II.A.3 in Cueva de Ardales. However, the authors believe this date to be incorrect, as ^14^C dating from the same CaCO_3_ sample yielded a considerably younger age (between 33,769 and 27,491 cal BP). They admit that both dating techniques, U/Th and radiocarbon, may deliver erroneous dates due to contamination of the samples but prefer the younger date. In Nerja, the Uranium leaching process, a possible source of error, cannot be ruled out because a single sample was taken for U/Th dating (note that in Cueva de Ardales a series of samples was taken along the growth axis of the calcite layer). Concerning the radiocarbon dates, bacterial CO_2_ fixation may be a source of significant error. Also, contamination of samples during handling (i.e. addition of minimal modern atmospheric carbon into the sample) may lead to unsound erroneous results. In order to precisely determine the chronology of its rock art further datings should be undertaken in Nerja (see comment by Zilhao) [35]. The reassessment of these dates would certainly shed light on the very contentious issue of the chronology of non-figurative Palaeolithic rock art in Iberia.

Considering the abstract paintings in a broader context, it must be taken into account that this kind of dots and lines are the most common motifs in European Palaeolithic rock art [36, 37]. Despite this, they have not been paid the attention they deserve and take a back seat in scientific studies. Yet, they probably reflect a very long tradition of marking cave walls beginning in the Middle Palaeolithic, or even earlier, and through to the Upper Palaeolithic or even younger phases.

## Conclusions

A series of 50 radiocarbon and 12 U/Th dates obtained within the framework of the archaeological excavation confirms a long history of human occupation in Cueva de Ardales (Fig 15) [53, 54]. Over 60 U/Th taken from calcite samples covering rock art at the cave gave very valuable additional chronological information. According to these dates, Neanderthals entered the cave in the Middle Palaeolithic, over 65,000 years ago. They left traces of symbolic practices on the cave walls and of tool maintenance in zones 3 and 5. Thereafter the cave was repeatedly visited by humans all the way to the late Neolithic/Chalcolithic period. However, the excavation has also revealed long hiatuses in human activity. The earliest one begins after the Middle Palaeolithic and is a common feature in the Palaeolithic record of Southern Iberia. An Aurignacian occupation cannot be proven with certainty in Ardales. It is not until the Gravettian that we can again be confident of the presence of humans in zone 5 and zone 2. The Solutrean is represented by one late radiocarbon date in zone 2 and is otherwise only sparsely represented in the lithic assemblages found in the excavation area. This could be related to the fact that substantial sectors of zone 2 are disturbed or relocated. A diagnostic leaf point found out of context on the floor, supports the presence of Solutrean groups in the cave. Some indication of Magdalenian and Epipalaeolithic occupations provided by isolated radiocarbon dates, but no more substantial evidence could be found in the excavated layers. This agrees with site frequency and distribution in Southern Iberia, which is characterized by a decrease in the number of sites during the Magdalenian. It was probably not until the Early Neolithic that the cave was again intensively visited. Finds from the niche next to the stairs reveal the use of the cave as a burial place. This is also attested by the presence of human bones found scattered on the surface in various sectors of the cave and in zone 2, and by preliminary finds from Galerías Altas, a separated cave system above the excavated areas of the Galerías Bajas. The future study and excavation of the Galerías Altas will complement these results.

The quantity and nature of the materials identified during the excavations indicate that Cueva de Ardales was not a campsite, but was only visited to carry out non-domestic tasks. During the Palaeolithic, the cave was certainly used for the production of rock art, which is attested by the presence of more than 1,000 motifs and the presence of a number of potential lumps of ochre in the excavated context. This non-domestic use of the cave continues later in the Neolithic and Chalcolithic, when the cave was used as burial place.
